# Integrating Early Tuberculosis States Into Contact Management in Peru

**DOI:** 10.1001/jamanetworkopen.2025.25207

**Published:** 2025-08-06

**Authors:** Qi Tan, Chuan-Chin Huang, Alicia Madden, Megan B. Murray

**Affiliations:** 1 Department of Global Health and Social Medicine, Harvard Medical School, Boston, Massachusetts; 2 Division of Global Health Equity, Department of Medicine, Brigham and Women’s Hospital, Boston, Massachusetts; 3 Department of Epidemiology, Harvard T. H. Chan School of Public Health, Boston, Massachusetts

## Abstract

**Importance:**

Tuberculosis (TB) is now understood to exist on a spectrum from TB infection to active TB disease. While World Health Organization guidelines target TB infection and TB disease, they overlook individuals with early TB in the middle of this spectrum.

**Objective:**

To evaluate chest radiograph (CR)–guided screening strategies among household contacts (HHCs) of patients with TB in a high-burden setting.

**Design, Setting, and Participants:**

This decision analytical model was constructed from June 1 to November 31, 2024. Community-based care in an environment with high TB burden and limited resources was used as the setting in Lima, Peru. Participants included a hypothetical cohort of 1000 HHCs with positive results of a tuberculin skin test and negative results of a sputum culture who were cleared of TB disease based on a clinician’s evaluation. The hypothetical cohort was based on the clinical and demographic features of a cohort studied between September 1, 2009, and August 29, 2012.

**Interventions:**

Strategy 1 included CR screening to rule out TB disease followed by TB preventive therapy for all; strategy 2, CR screening with treatment for TB disease for those with abnormal CR findings and TB preventive therapy for those without; and strategy 3, observation without pharmacological intervention. We modeled 6 intervention scenarios by applying strategies 1 and 2 to the entire HHC population, to HHCs younger than 35 years, and to HHCs younger than 19 years (Peru’s national TB policy).

**Main Outcomes and Measures:**

TB cases averted, serious adverse events (SAEs), and drug resistance.

**Results:**

A simulated cohort of 1000 HHCs with age and clinical status distributions was based on a previously published cohort of 12 767 TB HHCs in Lima, Peru. Of these, 7661 (60.0%) were male, 4212 (33.0%) were younger than 15 years, and 444 (3.4%) developed TB during 1 year of follow-up. Strategy 2 applied to all HHCs reduced TB cases by 71% to 81%, outperforming strategy 1 applied to all HHCs, which reduced cases by 49% to 69%. Strategy 2 reduced acquired isoniazid resistance but increased SAEs. When both strategies were restricted to HHCs younger than 19 years, strategy 2 reduced TB cases by 42% to 50%. Expanding treatment to older adults further reduced cases but also increased SAEs (19-22 additional SAEs).

**Conclusions and Relevance:**

In this model of TB HHC management, CR-guided identification and treatment of early TB was more effective than universal isoniazid preventive therapy, especially in children and young adults. Trade-offs between benefit and harm must be carefully considered in older adults.

## Key Points

**Question:**

In settings with high tuberculosis (TB) burden, should a household contact management strategy using chest radiograph (CR)–guided treatment of early TB be favored over universal TB preventive therapy as recommended by the World Health Organization?

**Findings:**

In this decision analytical model based on 12 767 household contacts in Lima, Peru, CR-guided identification and treatment of early TB with a regimen for TB disease was more effective than universal TB preventive therapy, although it was associated with more serious adverse events, particularly among older adults.

**Meaning:**

These findings suggest that CR-guided treatment for early TB is preferable for children and young adults in high-burden settings, with cautious consideration of serious adverse event risk in older populations.

## Teaser

This decision analytical model evaluates strategies for managing household contacts of patients with tuberculosis (TB) who have abnormal chest radiograph findings but do not meet clinical criteria for TB disease.

## Introduction

Tuberculosis (TB) remains one of the leading causes of death from an infectious disease worldwide.^1^ Despite decades of progress, efforts to interrupt TB transmission continue to fall short in many high-burden settings.^2,3^ One of the most promising strategies for reducing transmission is expanding access to TB preventive therapy (TPT) for household contacts (HHCs) of index patients with TB.^4,5^ While the World Health Organization (WHO) previously recommended TPT only for child HHCs younger than 5 years,^6^ recent guidelines have broadened eligibility to include all HHCs with positive test results for TB infection by either a tuberculin skin test (TST) or an interferon-gamma release assay.^7^

Before TPT can be safely given, however, WHO guidance emphasizes the importance of ruling out active TB disease.^6,7^ Offering TPT to someone with undiagnosed TB risks delaying appropriate treatment and increasing the chances of poor outcomes and drug resistance.^8,9^ The current recommendation is to screen eligible individuals using 1 or more of 3 tools: a 4-symptom screen (cough, weight loss, night sweats, and fever), radiograph (CR), and molecular testing.^10^ In many low-resource settings, this screening often relies on symptom assessment alone due to limited access to radiography or diagnostics.^11^

Although this framework—distinguishing TB infection from TB disease—provides operational clarity, it overlooks the spectrum between infection and symptomatic, microbiologically confirmed TB.^12,13^ Various terms—subclinical, asymptomatic, and incipient—have been used to describe this early disease state.^14^ As yet, there is little guidance on how to manage individuals who fall into this category,^8,15^ especially in settings where diagnostic tools are limited and the risks of overtreatment or undertreatment are high.^11,16^

In previous work, researchers including some of us found that among HHCs who were TST-positive and ruled out for TB disease based on clinical evaluation, those with abnormal CR findings were at significantly higher risk of developing TB during follow-up.^17,18^ Among children in this group, those who received TPT were strongly protected against progression compared with those who did not.^17^ These findings suggest that CRs can help identify individuals with early TB who fall between latent infection and overt disease and raise critical questions about how best to treat them. In this study, we used decision modeling to evaluate strategies for managing HHCs with abnormal CRs who did not meet clinical criteria for TB disease. Our goal was to inform more nuanced, risk-based approaches to prevention that took early TB states into account and to tailor this assessment to the specific setting of Lima, Peru, an urban setting with a moderately high TB burden in which current policy targets HHCs younger than 19 years.

## Methods

The cohort study that provided the demographic and clinical data for the decision analytic model was reviewed and approved by the Institutional Review Board of Harvard School of Public Health and the Research Ethics Committee of the National Institute of Health of Peru. We followed the Consolidated Health Economic Evaluation Reporting Standards (CHEERS) guidelines. Model analyses were conducted from June 1 to November 30, 2024.

### Simulated Cohort and Definition of Early TB

We developed a simulated cohort based on the age and clinical characteristics of a previously studied group of 12 767 HHCs of newly diagnosed patients with culture-positive TB in Lima, Peru. Peru is one of the 30 high-burden countries for TB identified by the WHO.^19^ In 2023, the national TB incidence in Peru was estimated at 173 cases per 100 000 population per year.^1^ Baseline characteristics of this population are summarized in eTable 1 in Supplement 1. We considered individuals to have early TB if they (1) were TST-positive HHCs of patients with TB, (2) had negative mycobacterial culture yields, (3) were ruled out for TB disease based on a clinical history and examination findings, and (4) had any abnormal findings on CR.^17,18,20^

### Intervention Strategies

**Figure 1.**
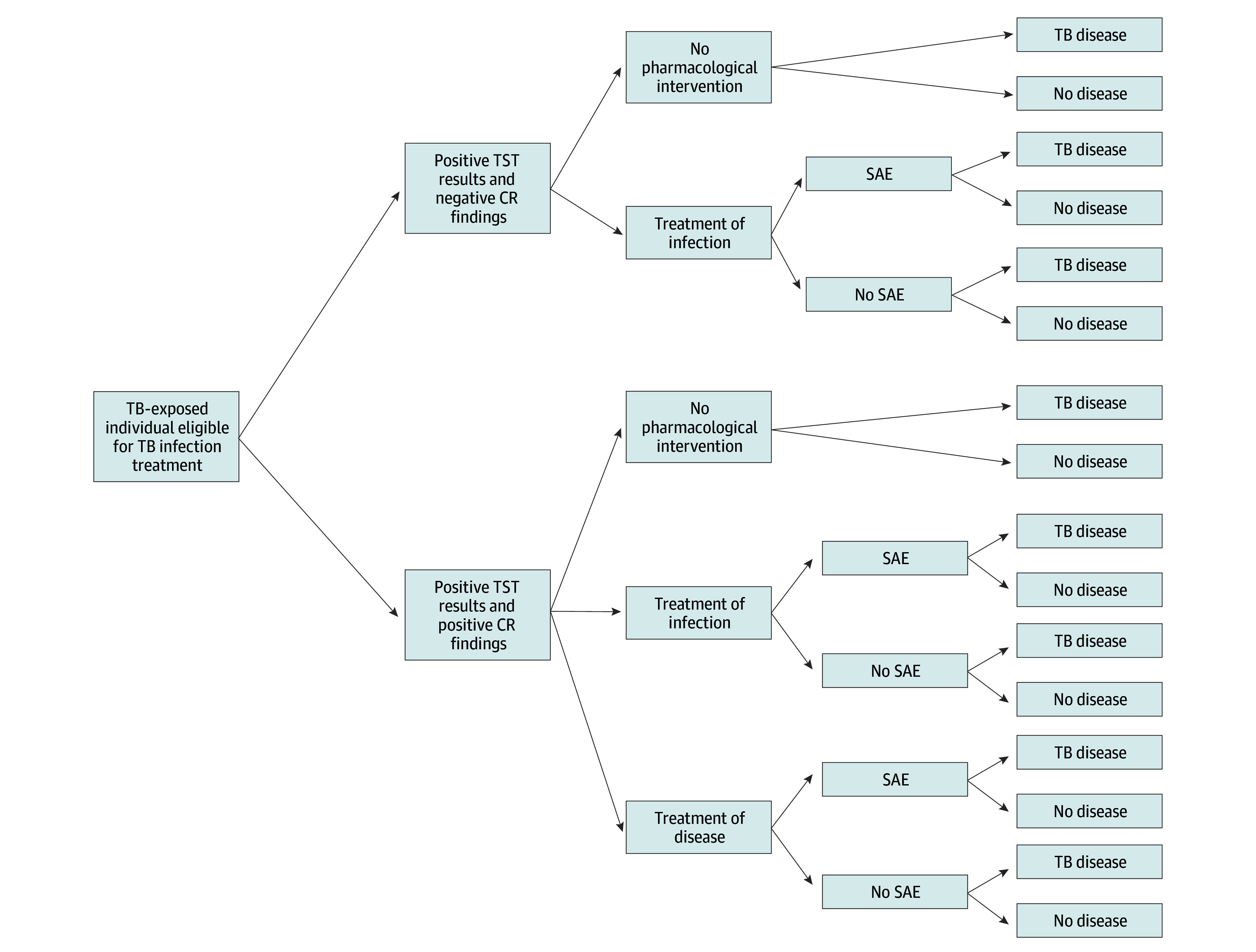
Decision Tree Framework of Contact Management Based on Baseline Chest Radiograph (CR) Results SAE indicates severe adverse events; TB, tuberculosis; and TST, tuberculin skin test.

To evaluate the potential benefits and harms of incorporating baseline CR findings into the management of TST-positive HHCs who had been ruled out for active TB disease, we modeled 3 strategies. Strategy 1 consisted of universal TPT (CR screening to rule out TB disease followed by prevention). All HHCs in whom TB disease was ruled out received TPT, regardless of CR results. Strategy 2 included CR-guided treatment (CR screening and treatment for early TB). HHCs with normal CR findings received TPT; those with abnormal CR findings were treated with a full TB disease regimen. Strategy 3 consisted of observation without pharmacological intervention. These strategies reflected distinct clinical decision points based on baseline CR interpretation (Figure 1). We further stratified these interventions based on the ages of the target population. Although the WHO conditionally recommends TPT for all adult HHCs,^7^ its routine use in older adults remains controversial due to elevated SAE risk.^21,22^ Peru’s national TB policy currently limits TPT to contacts younger than 19 years.^23,24^ Given these considerations, we applied each strategy to the full cohort and to 3 age-specific subgroups, yielding 6 scenarios: strategy 1 applied to all HHCs (1A); strategy 1 limited to HHCs younger than 35 years (1B); strategy 1 limited to HHCs younger than 19 years (1C; aligned with Peru’s national policy); strategy 2 applied to all HHCs (2A); strategy 2 limited to HHCs younger than 35 years (2B); and strategy 2 limited to contacts younger than 19 years (2C).

### Decision Tree Model

We used a decision tree framework to simulate individual-level outcomes under each strategy. For each branch, we modeled the probability of developing TB disease within 1 year, stratified by CR findings, age, and intervention type. We also incorporated the risk of SAEs, defined as grade 3 or higher according to clinical trial criteria,^25^ and assumed that individuals who experienced SAEs discontinued therapy and derived no protective benefit.^26,27^

For individuals with abnormal CR findings not considered diagnostic of TB disease, we modeled 3 options: TPT (A1), TB disease treatment (A2), and no pharmacological intervention (A3). For individuals with normal CR findings, we modeled TPT (B1) and no pharmacological intervention (B2). These arms allowed us to evaluate both benefits (TB cases averted) and harms (SAEs or drug resistance) under each intervention strategy, across all age groups.

We modeled 3 strategies: (1) isoniazid preventive therapy (IPT) for all (A1 and B1), (2) CR-guided treatment (A2 and B1), and (3) nonpharmacological intervention (A3 and B2). We projected incidence of TB disease and SAEs as primary outcomes and measured TB cases averted, number needed to treat, number needed to harm, and acquisition of drug resistance associated with unsuccessful TPT.

#### Treatment of Infection

We evaluated 3 TPT regimens: 6 months of daily isoniazid (IPT), 3 months of weekly isoniazid and rifapentine (3HP), and 4 months of daily rifampicin (4R). While all 3 regimens have shown similar effectiveness in preventing TB disease among individuals with TB infection,^27,28,29^ there are no data on the efficacy of 3HP and 4R in early TB. Compared with IPT, both 3HP and 4R are associated with higher treatment completion rates and lower risk of hepatotoxicity.^26,30^

#### Treatment of TB Disease

We modeled treatment of TB disease in individuals with early TB disease as follows. For children younger than 15 years, 2 months of isoniazid, rifampin, pyrazinamide, and ethambutol, followed by 2 months of isoniazid and rifampin (2HRZE/2HR), as recommended by the WHO for nonsevere TB, was followed.^31,32^ For those older than 15 years, the standard 6-month regimen of 2 months of isoniazid, rifampin, pyrazinamide, and ethambutol, followed by 4 months of isoniazid and rifampin (2HRZE/4HR), as per WHO guidelines, was followed.^32^

### Model Parameters

eTable 2 in Supplement 1 summarizes the key modeling parameters. We obtained parameter estimates (eTable 3 in Supplement 1) through moment matching and incorporated uncertainty by applying appropriate probability distributions. Point estimates and their corresponding distributions are illustrated in the Table and eFigure 1 in Supplement 1.

**Table.** Model Parameters

Scenario by source	Intervention arm	Age group, y	Estimate (IQR)
**Probability of progression to TB disease among TST-positive HHCs in whom TB has not been diagnosed**			
Baseline CR indicates normal results			
Huang et al,^17^ 2022	None	0-14	0.045 (0.038-0.053)
Tan et al,^18^ 2024	None	15-34	0.020 (0.016-0.023)
Tan et al,^18^ 2024	None	35-64	0.012 (0.01-0.014)
Tan et al,^18^ 2024	None	≥65	0.012 (0.01-0.014)
Baseline CR indicates abnormal results			
Huang et al,^17^ 2022	None	0-14	0.588 (0.507-0.668)
Tan et al,^18^ 2024	None	15-34	0.407 (0.356-0.457)
Tan et al,^18^ 2024	None	35-64	0.100 (0.078-0.122)
Tan et al,^18^ 2024	None	≥65	0.123 (0.073-0.174)
**Risk ratio of TB disease after IPT**			
Baseline CR screening indicates normal results			
Huang et al,^17^ 2022	IPT (6 mo)	0-14	0.309 (0.220-0.399)
Zenner et al,^33^ 2017 (network meta-analysis)	IPT (6 mo)	15-34	0.485 (0.416-0.554)
Zenner et al,^33^ 2017 (network meta-analysis)	IPT (6 mo)	35-64	0.485 (0.416-0.554)
Zenner et al,^33^ 2017 (network meta-analysis)	IPT (6 mo)	≥65	0.485 (0.416-0.554)
Baseline CR screening indicates abnormal results			
Huang et al,^17^ 2022	IPT (6 mo)	0-14	0.185 (0.066-0.304)
Gray et al,^34^ 2023 (meta-analysis)	IPT (6 mo)	15-34	0.601 (0.475-0.727)
Gray et al,^34^ 2023 (meta-analysis)	IPT (6 mo)	35-64	0.601 (0.475-0.727)
Gray et al,^34^ 2023 (meta-analysis)	IPT (6 mo)	≥65	0.601 (0.475-0.727)
**Risk ratio of WHO recommended standard TB treatment regimen**			
Baseline CR screening indicating abnormal results			
Turkova et al,^31^ 2022 (SHINE trial)	2HRZE/2HR (4 mo)	0-14	0.030 (0.026-0.035)
Dorman et al,^35^ 2021 (Study 31/A5349) and Prajapati et al,^36^ 2023	2HRZE/4HR (6 mo)	15-34	0.079 (0.071-0.086)
Dorman et al,^35^ 2021 (Study 31/A5349) and Prajapati et al,^36^ 2023	2HRZE/4HR (6 mo)	35-64	0.111 (0.099-0.123)
Dorman et al,^35^ 2021 (Study 31/A5349) and Prajapati et al,^36^ 2023	2HRZE/4HR (6 mo)	≥65	0.185 (0.127-0.243)
Probability of a severe adverse event while receiving IPT			
Menzies et al,^28^ 2018	IPT (6 mo)	0-14	0.002 (0.001-0.003)
Campbell et al,^30^ 2020	IPT (6 mo)	15-34	0.015 (0.013-0.017)
Campbell et al,^30^ 2020	IPT (6 mo)	35-64	0.028 (0.025-0.031)
Campbell et al,^30^ 2020	IPT (6 mo)	≥65	0.053 (0.039-0.067)
Probability of a severe adverse event while receiving 4R			
Melnychuk et al,^26^ 2023	4R	0-14	0
Melnychuk et al,^26^ 2023	4R	15-34	0.009 (0.007-0.011)
Melnychuk et al,^26^ 2023	4R	35-64	0.009 (0.007-0.010)
Melnychuk et al,^26^ 2023	4R	≥65	0.018 (0.011-0.026)
Probability of a severe adverse event while receiving 3HP			
Melnychuk et al,^26^ 2023	3HP	0-14	0.005 (0.003-0.007)
Melnychuk et al,^26^ 2023	3HP	15-34	0.019 (0.017-0.021)
Melnychuk et al,^26^ 2023	3HP	35-64	0.037 (0.034-0.040)
Melnychuk et al,^26^ 2023	3HP	≥65	0.062 (0.05-0.076)
Probability of a severe adverse event while receiving standard multidrug routine			
Turkova et al,^31^ 2022 (SHINE trial)	2HRZE/2HR (4 mo)	0-14	0.078 (0.071-0.085)
Yee et al,^37^ 2003	2HRZE/4HR (6 mo)	15-34	0.053 (0.043-0.063)
Yee et al,^37^ 2003	2HRZE/4HR (6 mo)	35-64	0.087 (0.059-0.115)
Yee et al,^37^ 2003	2HRZE/4HR (6 mo)	≥65	0.149 (0.101-0.196)
Probability of acquiring isoniazid resistance among those who develop TB after receiving IPT among HHCs with abnormal baseline CRs			
Balcells et al,^9^ 2006	IPT (6 mo)	All	0.025 (0.013-0.44)
Asencios et al,^38^ 2009			

Abbreviations: 2HRZE/2HR, 2 months of isoniazid, rifampin, pyrazinamide, and ethambutol, followed by 2 months of isoniazid and rifampin; 2HRZE/4HR, 2 months of isoniazid, rifampin, pyrazinamide, and ethambutol, followed by 4 months of isoniazid and rifampin; 3HP, 3 months of weekly isoniazid and rifapentine; 4R, 4 months of daily rifampicin; CR, chest radiograph; HHC, household contact; IPT, 6-month daily isoniazid preventive therapy; TB, tuberculosis; TST, tuberculin skin test; WHO, World Health Organization.

To model IPT efficacy for children aged 0 to 15 years, we applied data from the previous Peruvian cohort study.^17^ For adults with abnormal CR findings, we used a meta-analysis of randomized clinical trials evaluating IPT efficacy.^34^ For adults with normal CR findings, we used efficacy estimates from a network meta-analysis of randomized clinical trials.^33^

To model TB disease treatment efficacy for children with abnormal CR findings, we used data from the SHINE (Shorter Treatment for Minimal Tuberculosis in Children) trial,^31^ which evaluated the WHO-recommended 4-month regimen for children younger than 17 years with nonsevere TB.^32^ We conservatively assumed that children with abnormal CR findings who developed TB within 1 year were experiencing early TB, although such abnormalities may reflect other conditions. For adults, we modeled TB disease treatment efficacy using data from Study 31/A5349^35^ and age-stratified estimates from an Indian cohort.^36^

SAE risk estimates for IPT were drawn from multiple sources. For children receiving 6-month IPT, we used a phase 3 pediatric trial^39^ and assumed comparability to 9-month IPT, as 90% of SAEs occur within 6 months.^40^ For adults, we used data from Campbell et al,^30^ assuming SAE risk was similar across CR categories. For children and adults receiving TB disease treatment, we used safety data from the SHINE trial^31^ and an observational study by Yee et al,^37^ respectively. In our analysis of newer TPT regimens, we used age-stratified SAE data for 4R and 3HP from a meta-analysis of SAE rates for TB infection,^26^ assuming efficacy for these regimens is equivalent to IPT for subclinical TB^17,34^ (eTable 2 in Supplement 1).

While IPT has not been shown to increase resistance at the population level,^9,41^ the emergence of resistance due to inadvertent monotherapy in individuals with unrecognized TB, particularly in low-resource settings is a concern.^8,16^ To estimate this risk, we combined data from a 2006 meta-analysis of 13 studies evaluating IPT-associated resistance^9^ with Peru’s 2005-2006 national surveillance data on baseline isoniazid resistance^38^ (eMethods in Supplement 1). We did not estimate resistance risk for newer TPT regimens, as no relevant data are currently available.

In the base-case analysis, we assumed 100% uptake of TPT. To better reflect clinical implementation, we also modeled uptake rates of 25%, 50%, and 75%, applying these uniformly across age groups and CR findings. In the Peru HHC cohort, only 49% of eligible individuals younger than 19 years initiated IPT^17^ (eTable 1 in Supplement 1). Reliable uptake data for adults were not available, and treatment completion rates were not reported.

### Statistical Analysis

#### Simulation 

We used a Monte Carlo simulation framework to project outcomes in a hypothetical cohort of 1000 TST-positive HHCs, incorporating the age distribution and prevalence of abnormal CRs observed in the prior cohort study^17,18^ (eTable 1 in Supplement 1). For each modeled scenario, we sampled 10 000 parameter sets from appropriate probability distributions to generate uncertainty estimates, reported as IQRs (eMethods in Supplement 1).

#### Model Output

For each strategy, we modeled the 1-year risks of incident TB disease and SAEs, accounting for the joint distribution of age- and CR-specific TB progression risk and the risk ratios associated with each intervention. For each implementation scenario, we estimated the expected number of TB cases and SAEs under each intervention, as well as the reduction in TB cases for each pharmacological intervention compared with the no-treatment scenario, expressed per 1000 HHCs. We also estimated the expected number of TB cases with acquired isoniazid monoresistance among individuals receiving IPT. All modeling and analyses were conducted using R, version 4.2.1 (R Program for Statistical Computing).

## Results

We simulated outcomes in a cohort reflecting the demographic and clinical characteristics of 12 767 HHCs of patients with culture-positive TB in Lima, Peru. In the reference cohort, 5106 patients (40.0%) were female, 7661 (60.0%) were male, and 4212 (33.0%) were younger than 15 years. During 1 year of follow-up, 444 individuals (3.5%) developed incident TB. Among all HHCs, 5313 (41.6%) had positive TST results and 2913 (22.8%) received IPT (eTable 1 in Supplement 1).

### TB Incidence, SAEs, and Risk-Benefit Estimates by Age and CR

**Figure 2.**
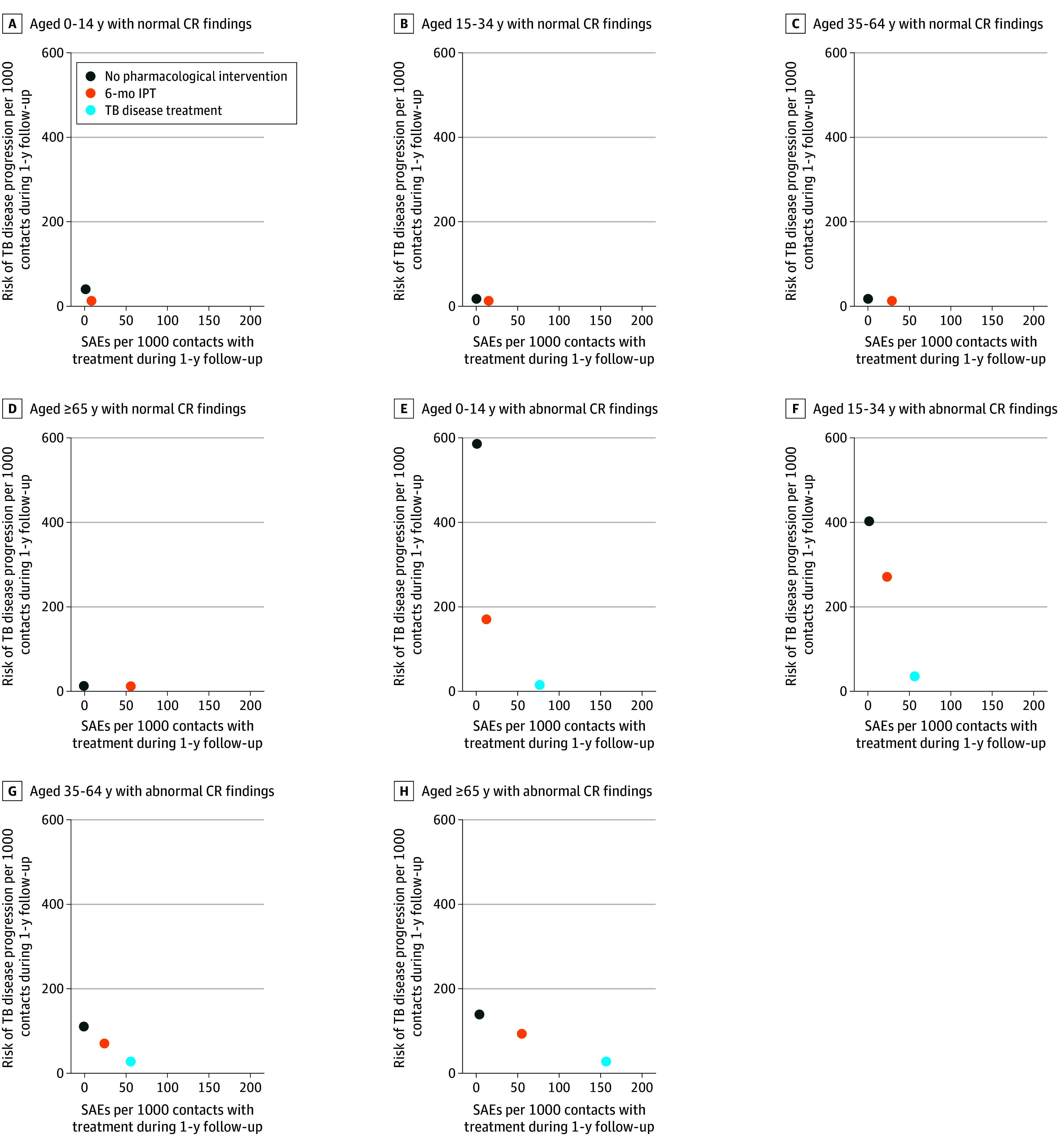
Estimated 1-Year Risk of Tuberculosis (TB) Progression and Severe Adverse Events (SAEs) by Age, Regimen, and Chest Radiograph (CR) Results IPT indicates isoniazid preventive therapy.

Figure 2 shows estimated 1-year TB and SAE risks by age and CR findings. Among TST-positive HHCs with abnormal CR findings, the 1-year TB risk without treatment was highest in children and declined with age—from 588 (IQR, 505-673) cases per 1000 HHCs in children aged 0 to 14 years to 133 (IQR, 75-178) cases per 1000 HHCs in adults older than 65 years. In children, TB incidence dropped to 166 (IQR, 55-200) cases per 1000 HHCs with IPT and to 18 (IQR, 14-21) cases per 1000 HHCs with TB disease treatment. Across all age groups, TB disease treatment was more effective than isoniazid in reducing TB incidence among those with abnormal CR findings.

For HHCs with normal CR findings, TB incidence was lower, but SAE risk increased with age for IPT. Among TPT options, 4R had the lowest SAE risk, and 3HP the highest. Details are summarized in eTable 4 in Supplement 1.

### Comparison of Intervention Strategies by Age Group

**Figure 3.**
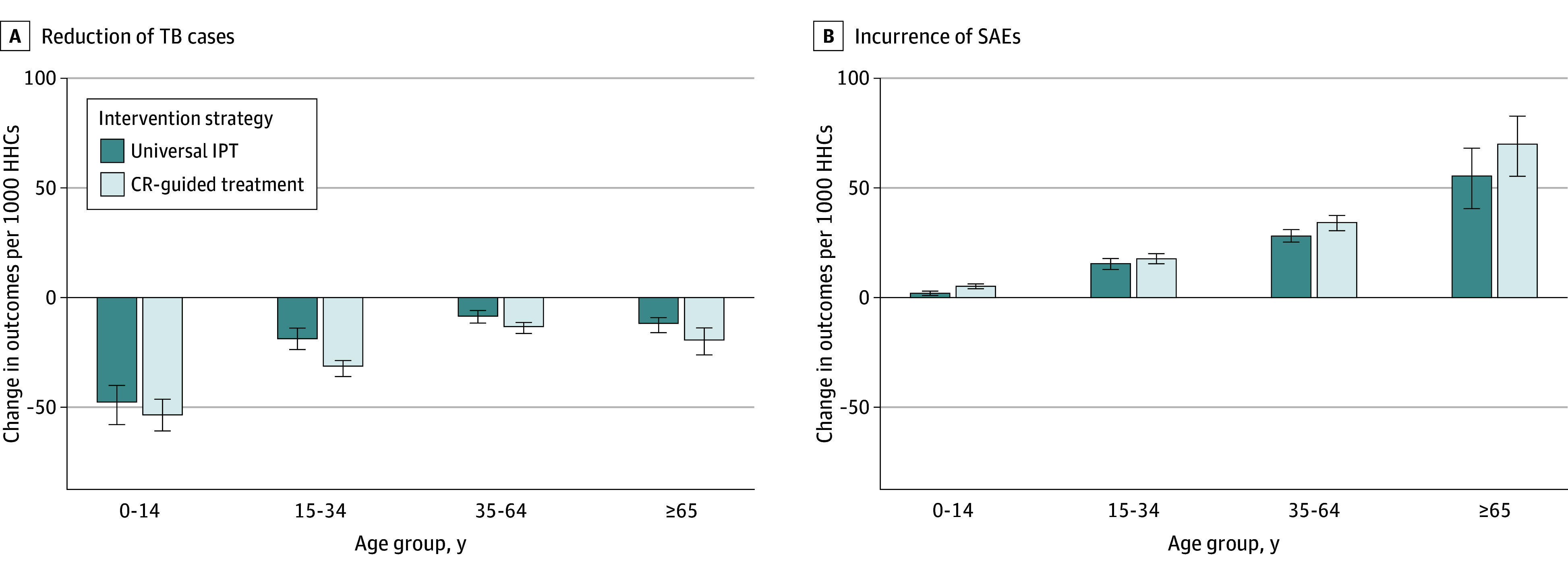
Comparison of Management Strategies Across Age Groups Prevalence of chest radiograph (CR) abnormal findings among household contacts (HHCs) free of tuberculosis (TB) disease are based on EPI cohort of previous studies^17,18^: for those aged 0 to 14 years, 4%; 15 to 34 years, 6%; 35 to 64 years, 9%; and 65 years or older, 14%. Bars represent median number of cases vs nonpharmacological scenario per 1000 contacts by age group. Error bars represent IQRs. IPT indicates isoniazid preventive therapy; and SAEs, severe adverse events.

Figure 3 summarizes TB cases averted and SAEs across the 3 strategies by age. Strategy 2 (CR-guided treatment) reduced more TB cases than strategy 1 (universal IPT) but incurred more SAEs. For every 1000 children younger than 15 years, strategy 1 prevented 47 (IQR, 39-58) TB cases with 2 (IQR, 1-3) SAEs, while strategy 2 prevented 53 (IQR, 46-61) TB cases with 5 (IQR, 4-6) SAEs. In adults aged 35 to 64 years and 65 years or older, both TB reduction and SAEs shifted, with higher SAE incidence. Details are in eTable 5 in Supplement 1.

### Impact of TPT Regimen

eFigure 2 and eTables 5 and 6 in Supplement 1 present outcomes for 6-month daily isoniazid, 4R, and 3HP by strategy and age. 4R yielded the lowest SAE incidence across groups. Scenario-specific projections for TB cases and SAEs using 4R and 3HP regimens are in eTables 9 and 10 in Supplement 1.

### Drug Resistance Risk

eFigure 3 in Supplement 1 illustrates higher rates of acquired isoniazid resistance under strategy 1 compared with strategy 2, particularly in younger groups. The overall highest risk occurred with strategy 1, at 0.6 (IQR, 0.2-1.0) cases per 1000 treated. Age-specific risks are in eTable 5 in Supplement 1.

### Outcomes Across All Intervention Scenarios

**Figure 4.**
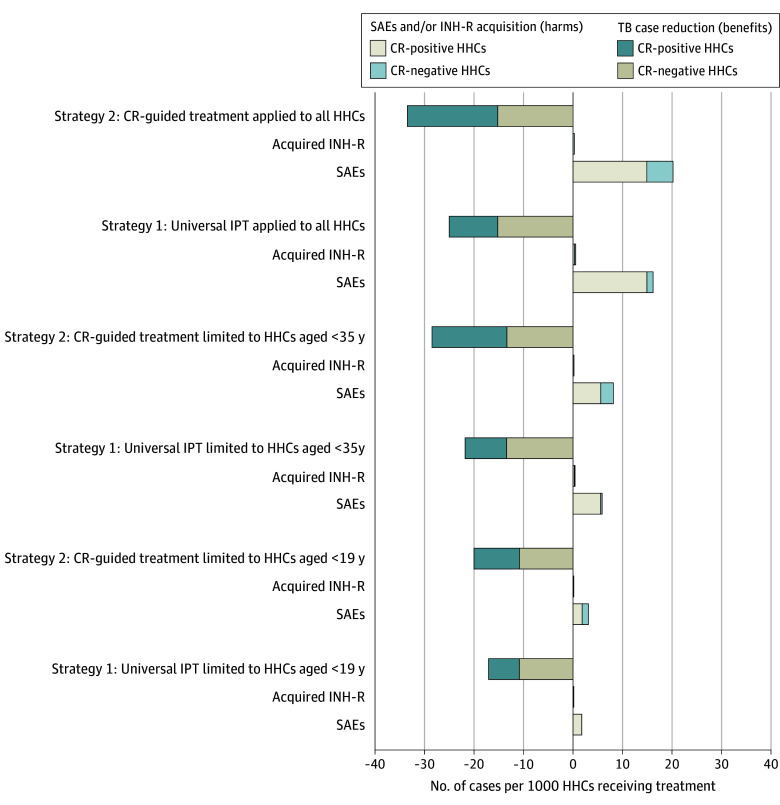
Estimated Benefits and Harms of 2 Management Strategies by Coverage Scenarios for Household Contacts (HHCs) in High-Burden Settings Applied parameters are from a high-burden country similar to Peru.^9,11^ Bars represent median number of cases vs nonpharmacological scenario. CR-negative indicates normal chest radiograph finding; CR-positive, abnormal CR finding; INH-R, isoniazid resistance; IPT, isoniazid preventive therapy; SAEs, severe adverse events; and TB, tuberculosis.

eTable 7 in Supplement 1 summarizes expected TB cases, SAEs, resistance, and risk-benefit estimates under each scenario. Without treatment, 44 (IQR, 40-47) TB cases per 1000 HHCs were projected. Scenario 2A (CR guided, all ages) reduced TB cases by 71% to 81% with 19 to 22 additional SAEs; scenario 1A (universal IPT) reduced TB by 49% to 69% with 15 to 18 SAEs. Under Peru’s current policy (scenario 1C), TB reduction was 34% to 47% with 1 to 2 SAEs, compared with 42% to 50% reduction and 3 to 4 SAEs in scenario 2C. Figure 4, eFigure 4, and eFigure 5 in Supplement 1 visualize these trade-offs. Expanding CR-guided treatment to older adults improved TB reduction and resistance mitigation but increased SAEs.

### Treatment Uptake Rates

eFigure 6 and eTable 8 in Supplement 1 show outcomes under 25%, 50%, 75%, and 100% treatment uptake rates. At 50% uptake, benefits and harms were diminished for all strategies. Further results appear in the eResults in Supplement 1.

## Discussion

In this decision analytical model, we found that treating HHCs with early TB—defined as those with an abnormal CR finding but cleared of TB after clinical assessment—with a full TB disease regimen was more effective than providing them with IPT. However, it also resulted in a higher incidence of SAEs, especially in older adults. The universal IPT strategy was associated with a higher risk of acquired isoniazid resistance, particularly among younger populations. These findings support the integration of CR findings into HHC management to better target individuals with early disease.

In addition to IPT, we evaluated 2 newer rifamycin-based TPT options that are increasingly available and offer potential advantages over IPT. In our model, 4R was associated with the lowest incidence of SAEs and may be the preferred option, particularly in older populations. In contrast, 3HP had the highest SAE incidence. Assuming similar efficacy across regimens for early TB, these findings support prioritizing 4R in high-risk groups while emphasizing the need for age-stratified safety assessments in future studies.

Age-targeted strategies substantially influenced the risk-benefit balance. In children and young adults, both the CR-integrated intervention strategy and universal TPT demonstrated more favorable efficacy in reducing TB cases, while the risk of SAEs remained low across both approaches. However, in adults older than 65 years, the risk of SAEs increased significantly, reducing the net benefit. These results underscore the potential advantages of treating early TB before it progresses and highlight the need for selective treatment approaches in older adults.^21,22^

Implementation of a CR-guided strategy will require expanded diagnostic capacity.^11,14^ In low-resource settings such as Peru—where CR is not routinely performed for HHCs^24^—scaling up portable, artificial intelligence–assisted digital radiography could improve feasibility.^10^ In settings without access to imaging, test-free or symptom-independent algorithms may offer broader coverage but carry greater risk of adverse outcomes in older adults.^42,43^ Uptake of and adherence to both TPT and treatment of TB disease remain crucial and notoriously hard to achieve in people without symptoms of TB disease^44,45^; in our model, lower uptake rates reduced overall impact but also mitigated SAEs.

### Limitations

This study has several limitations. First, the effectiveness of TPT may have been underestimated due to contextual factors specific to our setting. In communities with moderate TB prevalence, HHCs may be reinfected through community exposure after receiving TPT, diminishing its protective effect.^46^ A high burden of coprevalent TB within households may further reduce the incremental benefit of TPT for preventing incident disease. Our assumptions about TPT efficacy may also overlook the population-level effects of widespread implementation in stable communities, where sustained uptake could reduce transmission.

Second, we modeled a hypothetical cohort based on data from Peru—a country with moderate TB incidence and low HIV prevalence.^19^ These factors may limit generalizability to settings with different epidemiologic profiles, particularly those with high rates of HIV or multidrug-resistant TB. Our model did not include HIV-positive contacts or account for potential differences in drug resistance patterns beyond isoniazid monoresistance.

Third, our model applied a uniform screening strategy across all age groups, despite known limitations of TST and CR in young children.^7,10^ Given the variability in TB presentation and diagnostic yield by age, age-specific or multimodal screening approaches may improve detection and outcomes and warrant further evaluation.

Last, the model did not account for broader benefits of early TB treatment, such as reduced transmission, prevention of post-TB lung disease, or stigma reduction. We assumed perfect implementation, including full adherence to testing and treatment protocols, and drew efficacy and safety estimates from clinical trial data. These assumptions may not fully reflect clinical programmatic challenges.

## Conclusions

In this decision analytical model of TB management strategies for HHCs, integrating CR-guided treatment for early TB states was projected to prevent more TB cases and reduce isoniazid resistance compared with universal TPT, particularly in younger groups. However, the risk of SAEs—especially in older adults—requires careful consideration. These findings support CR-guided management as a promising strategy for high-burden settings and highlight priorities for future research and implementation.

## Notes

Supplement 1. **eMethods.** Uncertainty Estimation, Parameters and Equations, and Details of Parameter Estimation **eTable 1.** Parameters From the Real Peruvian Household Contact Cohort **eResults.** Scenario Findings **eTable 2.** Source Data Extracted for Each Parameter **eTable 3.** Distributional Assumptions for Model Parameters **eTable 4.** One-Year Risk of TB and SAEs by Age, Regimen, and Initial CXR Results **eTable 5.** Estimated Outcomes and Differences Between 2 Contact Management Strategies by Age Group **eTable 6.** Estimated Outcomes and Differences Between 2 Intervention Strategies by Age Group Applying 3HP or 4R **eTable 7.** Outcomes per 1000 Contacts by Intervention Scenarios Using 6H as the TPT Regimen **eTable 8.** Outcomes per 1000 Contacts by Intervention Scenarios Using 6H as the TPT Regimen When Overall Uptake Rate Is 50% **eTable 9.** Outcomes per 1000 Contacts Under Intervention Scenarios Using 4R as the TPT Regimen **eTable 10.** Outcomes per 1000 Contacts Under Intervention Scenarios Using 3HP as the TPT Regimen **eFigure 1.** Plots of Probability Distribution Functions for Model Parameters in eTable 2 **eFigure 2. **Outcomes of 2 Pharmacological Strategies Across Age Groups Using Different TPT Regimens (6H, 4R, and 3HP) **eFigure 3. **Comparison of Isoniazid Resistance Acquisition Cases Between 2 Strategies by Age Group **eFigure 4. **Estimated Efficacy of 2 Strategies by 6 Intervention Scenarios Compared With Nonpharmacological Intervention Scenario **eFigure 5. **Comparing Outcomes of Scaling Up 2 Intervention Strategies to All Contacts When Applying 6H, 4R, and 3HP as TPT Regimen **eFigure 6. **Differences in Outcomes Between 2 Pharmacological Strategies Across Age Groups Under Varying Uptake Rate
eReferences.

Supplement 2.
Data Sharing Statement
