# Supplementary material for: Integrating Early Tuberculosis States Into Contact Management in Peru
Source: JAMA Netw Open. 2025 Aug 6;8(8):e2525207. doi: 10.1001/jamanetworkopen.2025.25207 (PMC12329612; doi:10.1001/jamanetworkopen.2025.25207)
Supplement: Supplement 1. — eMethods. Uncertainty Estimation, Parameters and Equations, and Details of Parameter Estimation eTable 1. Parameters From the Real Peruvian Household Contact Cohort eResults. Scenario Findings eTable 2. Source Data Extracted for Each Parameter eTable 3. Distributional Assumptions for Model Parameters eTable 4. One-Year Risk of TB and SAEs by Age, Regimen, and Initial CXR Results eTable 5. Estimated Outcomes and Differences Between 2 Contact Management Strategies by Age Group eTable 6. Estimated Outcomes and Differences Between 2 Intervention Strategies by Age Group Applying 3HP or 4R eTable 7. Outcomes per 1000 Contacts by Intervention Scenarios Using 6H as the TPT Regimen eTable 8. Outcomes per 1000 Contacts by Intervention Scenarios Using 6H as the TPT Regimen When Overall Uptake Rate Is 50% eTable 9. Outcomes per 1000 Contacts Under Intervention Scenarios Using 4R as the TPT Regimen eTable 10. Outcomes per 1000 Contacts Under Intervention Scenarios Using 3HP as the TPT Regimen eFigure 1. Plots of Probability Distribution Functions for Model Parameters in eTable 2 eFigure 2. Outcomes of 2 Pharmacological Strategies Across Age Groups Using Different TPT Regimens (6H, 4R, and 3HP) eFigure 3. Comparison of Isoniazid Resistance Acquisition Cases Between 2 Strategies by Age Group eFigure 4. Estimated Efficacy of 2 Strategies by 6 Intervention Scenarios Compared With Nonpharmacological Intervention Scenario eFigure 5. Comparing Outcomes of Scaling Up 2 Intervention Strategies to All Contacts When Applying 6H, 4R, and 3HP as TPT Regimen eFigure 6. Differences in Outcomes Between 2 Pharmacological Strategies Across Age Groups Under Varying Uptake Rate eReferences. [file jamanetwopen-e2525207-s001.pdf]

Supplementary Online Content

Tan Q, Huang C-C, Madden A, Murray MB. Integrating early tuberculosis states into contact management in Peru. *JAMA Netw Open.* 2025;8(8):e2525207. doi:10.1001/jamanetworkopen.2025.25207

eMethods. Uncertainty Estimation, Parameters and Equations, and Details of Parameter Estimation
eTable 1. Parameters From the Real Peruvian Household Contact Cohort
eResults. Scenario Findings
eTable 2. Source Data Extracted for Each Parameter
eTable 3. Distributional Assumptions for Model Parameters
eTable 4. One-Year Risk of TB and SAEs by Age, Regimen, and Initial CXR Results
eTable 5. Estimated Outcomes and Differences Between 2 Contact Management Strategies by Age Group
eTable 6. Estimated Outcomes and Differences Between 2 Intervention Strategies by Age Group Applying 3HP or 4R
eTable 7. Outcomes per 1000 Contacts by Intervention Scenarios Using 6H as the TPT Regimen
eTable 8. Outcomes per 1000 Contacts by Intervention Scenarios Using 6H as the TPT Regimen When Overall Uptake Rate Is 50%
eTable 9. Outcomes per 1000 Contacts Under Intervention Scenarios Using 4R as the TPT Regimen
eTable 10. Outcomes per 1000 Contacts Under Intervention Scenarios Using 3HP as the TPT Regimen
eFigure 1. Plots of Probability Distribution Functions for Model Parameters in eTable 2
eFigure 2. Outcomes of 2 Pharmacological Strategies Across Age Groups Using Different TPT Regimens (6H, 4R, and 3HP)
eFigure 3. Comparison of Isoniazid Resistance Acquisition Cases Between 2 Strategies by Age Group
eFigure 4. Estimated Efficacy of 2 Strategies by 6 Intervention Scenarios Compared With Nonpharmacological Intervention Scenario
eFigure 5. Comparing Outcomes of Scaling Up 2 Intervention Strategies to All Contacts When Applying 6H, 4R, and 3HP as TPT Regimen
eFigure 6. Differences in Outcomes Between 2 Pharmacological Strategies Across Age Groups Under Varying Uptake Rate
eReferences.

© 2025 Tan Q et al. *JAMA Network Open*.

This supplementary material has been provided by the authors to give readers additional information about their work.

© 2025 Tan Q et al. *JAMA Network Open*.

eMethods. Uncertainty Estimation, Parameters and Equations, and Details of Parameter Estimation

I. Uncertainty estimation

For uncertainty in probability (risk) parameters, we applied a beta distribution, as it is suitable for modeling percentages and probabilities, and serves as a conjugate prior probability distribution for the Bernoulli distribution[1,2].For the uncertainty in risk ratio parameters, we used a lognormal distribution. This choice is based on the standard epidemiological assumption that the natural log of the risk ratio follows a normal distribution[3], which implies that the risk ratio itself follows a lognormal distribution.

Equations used in estimation uncertainty in parameters are shown as below.

For probability (risk) of TB disease, probability (risk) of SAE and probability (risk) of acquired INH-Resistance, we applied beta distribution:

$$X \sim Beta(\alpha, \beta)$$

For risk ratio of intervention effect, we applied lognormal distribution:

$$X \sim \mathrm{lognormal}(\mu, \sigma 2)$$

Distribution for each parameter were listed in eTable 3. Plots of probability distribution functions for model parameters were shown in eFigure 1

II. Parameters and Equations used for projecting outputs

To describe how we combine these factors in our estimate in number of TB disease cases over one year, we introduced some additional terminology to represent each parameter as shown below:

For Parameter i=1 to 4,

i=1: HHC group aged 0-14 years.

i=2: HHC group aged 15-34 years.

i=3: HHC group aged 35-64 years.

i=4: HHC group aged over 65 years.

ei: The number of household contacts underwent CXR screening and eligible for TPT at baseline.

pi: The probability of developing incident TB over one year without receiving any intervention (nature history of TB) with an abnormal baseline CXR among a given age group eligible for TPT

ni: The probability of developing incident TB over one year without receiving any intervention (nature history of TB) with a normal baseline CXR among a given age group eligible for TPT

p6hi: The effect (risk ratio) on 6 month-IPT for the contacts with an abnormal baseline CXR in a given age group

n6hi: The effect (risk ratio) on 6 month-IPT for the contacts with a normal baseline CXR in a given age group

ti: The effect (risk ratio) on disease treatment regimen (HRZE) for the contacts with a normal baseline CXR in a given age group

S6hi: The probability of developing severe adverse events among contacts on 6 month-IPT regimen in a given age group, assuming the risk of developing SAEs on IPT for eligible contacts doesn't vary by baseline CXR status.

Sti: The probability of developing severe adverse events among contacts on HRZE regimen in a given age group

© 2025 Tan Q et al. *JAMA Network Open*.

ri: Proportion of screened household contacts eligible for TPT with an abnormal baseline CXR in a given age group.

1-ri : Proportion of TST positive contacts with a normal baseline CXR condition in the specific age group.

a: Probability of acquiring INH resistance if IPT is unsuccessful and an initially susceptible infection progresses to clinical disease among baseline CXR abnormal contacts across all age population. We estimate this for use of IPT among early TB states as of initial CXR abnormal population.

wi: Proportion of a specific age group among a whole household contact population across all ages in a high burden setting similar to Lima, Peru

We estimated the expected cases with natural progression to TB disease over one year among CXR-abnormal population in each age group per 1,000 contacts: 1,000*pi

We estimated the expected cases with natural progression to TB disease over one year among CXR-normal population in each age group per 1,000 contacts: 1,000*ni

We estimated the expected cases developing TB disease among CXR-abnormal population in each age group per 1,000 contacts treated by 6 month-IPT: 1,000*pi*(p6hi*(1-S6hi) +S6hi)

We estimated the expected cases developing TB disease among CXR-abnormal population in each age group per 1,000 contacts treated by active TB regimen: 1,000*pi*(ti*(1-Sti)+Sti)

We estimated the expected cases developing TB disease among CXR-normal population in each age group per 1,000 contacts treated by 6 month-IPT: 1,000*ni*(n6hi*(1-S6hi) +S6hi)

We estimated the expected cases developing SAE among CXR-abnormal or normal population in each age group per 1,000 contacts treated by 6 month-IPT: 1,000*S6hi

We estimated the expected cases developing SAE among CXR-abnormal population in each age group per 1,000 contacts treated by HRZE regimen: 1,000*Sti

We estimated the expected TB cases with acquired INH-resistance among CXR-abnormal population in each age group per 1,000 contacts treated by IPT: 1,000*ri*pi*(p6hi*(1-S6hi) +S6hi)*a

We estimated the expected contact number need to be treated i to avert one TB case n each age group with an abnormal baseline CXR if treated by IPT: $NNT_{CXR+} = \frac{1}{pi*(1-p6hi)}$

We estimated the expected contact number need to be treated to avert one TB case in each age group with an abnormal baseline CXR if treated by HRZE regimen: $NNT_{CXR+} = \frac{1}{pi*(1-ti)}$

We estimated the expected contact number need to be treated to avert one TB case in each age group with a normal baseline CXR if treated by IPT: $NNT_{CXR-} = \frac{1}{ni*(1-n6hi)}$

Based on these, we estimate the cumulative number of TB disease cases in each age group scenario per 1000 total population with baseline age stratified-CXR abnormality prevalence similar to a HHC population in Peru:

Expected overall TB cases over one year in each age group per 1,000 contacts in a nontreatment scenario:

© 2025 Tan Q et al. *JAMA Network Open*.

$$\sum_{TB\text{-}nontreati}=1{,}000*ri*pi+1{,}000*(1-ri)*ni$$

Expected overall TB cases over one year in each age group per 1,000 contacts in 'IPT-for-all' strategy scenario:

$$\sum_{TB\text{-}IPT\text{-}alli}=1{,}000*ri*pi*(p6hi*(1-S6hi)+S6hi)+1{,}000*(1-ri)*ni*(n6hi*(1-S6hi)+S6hi)$$

Expected reduced TB cases over one year in each age group per 1,000 contacts in 'IPT-for-all' strategy scenario:

$$\sum reduction_{-IPT\text{-}alli}=\sum_{TB\text{-}IPT\text{-}alli}-\sum_{TB\text{-}nontreati}$$

Expected overall SAE cases over one year in each age group per 1,000 contacts in 'IPT-for-all'strategy scenario:

$$\sum_{SAE\text{-}IPT\text{-}alli}=1{,}000*ri*S6hi+1{,}000*(1-ri)*S6hi$$

Expected overall acquired isoniazid resistance cases over one year in each age group per 1,000 contacts in 'IPT-for-all' strategy scenario:

$$\sum_{INHR\text{-}IPT\text{-}basedi}=a*\sum_{TB\text{-}IPT\text{-}alli}$$

Expected overall TB cases over one year in each age group per 1,000 contacts in 'CXR-based treatment' strategy scenario:

$$\sum_{TB\text{-}CXR\text{-}basedi}=1{,}000*ri*pi*(ti*(1-Sti)+Sti)+1{,}000*(1-ri)*ni*(n6hi*(1-S6hi)+S6hi)$$

Expected reduced TB cases over one year in each age group per 1,000 contacts in 'CXR-based treatment' strategy scenario:

$$\sum reduction_{-CXR\text{-}based\text{-}alli}=\sum_{TB\text{-}CXR\text{-}basedi}-\sum_{TB\text{-}nontreati}$$

Expected overall SAE cases over one year in each age group per 1,000 contacts in 'CXR-based treatment' strategy scenario:

$$\sum_{SAE\text{-}CXR\text{-}basedi}=1{,}000*ri*Sti+1{,}000*(1-ri)*S6hi$$

Expected overall acquired isoniazid resistance cases over one year in each age group per 1,000 contacts in 'CXR-based treatment' strategy scenario:

$$\sum_{INHR\text{-}CXR\text{-}basedi}=a*1{,}000*(1-ri)*ni*(n6hi*(1-S6hi)+S6hi)$$

Finally, we used the sample of the hypothetical HHC cohort to project the overall TB cases, overall TB case reduction, overall SAEs and overall cases with acquired isoniazid resistance despite IPT with each of the six-management strategy scenario, assuming e =1,000 across all age group in a HHC cohort, as below:

(0) Monitoring with non-treatment:

$$\sum_{TB\text{-}nontreat}=e*w1*\sum_{TB\text{-}nontreat1}+e*w2*\sum_{TB\text{-}nontreat2}+e*w3*\sum_{TB\text{-}nontreat3}+e*w4*\sum_{TB\text{-}nontreat4}$$

(1) IPT for all contacts

Overall TB cases:

$$\sum_{TB\text{-}IPT\text{-}ALL}=e*w1*\sum_{TB\text{-}IPT\text{-}all1}+e*w2*\sum_{TB\text{-}IPT\text{-}all2}+e*w3*\sum_{TB\text{-}IPT\text{-}all3}+e*w4*\sum_{TB\text{-}IPT\text{-}all4}$$

© 2025 Tan Q et al. *JAMA Network Open*.

Overall TB case reduction: $\sum_{TB\text{-}IPT\text{-}ALL}—\sum_{TB\text{-}nontreat}$

Overall SAEs:

$$\sum_{SAE\text{-}IPT\text{-}ALL}=e*(w1*S6h1+w2*S6h2+w3*S6h3+w4*S6h4)$$

Overall acquired INH-R cases:

$$\sum_{INHR\text{-}IPT\text{-}ALL}=a*\sum_{TB\text{-}IPT\text{-}ALL}$$

(2) CXR-based regimen for all contacts

Overall TB cases:

$$\sum_{TB\text{-}CXR\text{-}based}=e*w1*\sum_{TB\text{-}CXR\text{-}based1}+e*w2*\sum_{TB\text{-}CXR\text{-}based2}+ e*w3*\sum_{TB\text{-}CXR\text{-}based3}+e*w4*\sum_{TB\text{-}CXR\text{-}based4}$$

Overall TB case reduction: $\sum_{TB\text{-}CXR\text{-}based}—\sum_{TB\text{-}nontreat}$

Overall SAEs:

$$\sum_{SAE\text{-}CXR\text{-}based}=e*w1*(r1*St1+(1\text{-}r1)*S6h1)+e*w2*(r2*St2+(1\text{-}r2)*S6h2)+ e*w3*(r3*St3+(1\text{-}r3)*S6h3)+e*w4*(r4*St4+(1\text{-}r4)*S6h4)$$

Overall acquired INH-R cases:

$$\sum_{INHR\text{-}CXR\text{-}based}=a*(e*w1*((1\text{-}r1)*n1*(n6h1*(1\text{-}S6h1)+S6h1))+e*w2*((1\text{-}r2)*n2*(n6h2*(1\text{-}S6h2)+S6h2))+ e*w3*((1\text{-}r3)*n3*(n6h3*(1\text{-}S6h3)+S6h3))+e*w4*((1\text{-}r4)*n4*(n6h4*(1\text{-}S6h4)+S6h4)))$$

(3) IPT for contacts under 35 years only

Overall TB cases:

$$\sum_{TB\text{-}IPT<35}=e*w1*\sum_{TB\text{-}IPT\text{-}all1}+e*w2*\sum_{TB\text{-}IPT\text{-}all2}+ e*w3*\sum_{TB\text{-}nontreat3}+e*w4*\sum_{TB\text{-}nontreat4}$$

Overall TB case reduction: $\sum_{TB\text{-}IPT<35}—\sum_{TB\text{-}nontreat}$

Overall SAEs:

$$\sum_{SAE\text{-}IPT<35}=e*(w1*S6h1+w2*S6h2)$$

© 2025 Tan Q et al. *JAMA Network Open*.

Overall acquired INH-R cases:

$$\sum_{INHR\text{-}IPT<35}=a*\sum_{TB\text{-}IPT<35}$$

(4) CXR-based regimen for contacts under 35 years only

Overall TB cases:

$$\sum_{TB\text{-}CXR\text{-}based<35}=e*w1*\sum_{TB\text{-}CXR\text{-}based1}+e*w2*\sum_{TB\text{-}CXR\text{-}based2}+e*w3*\sum_{TB\text{-}nontreat3}+e*w4*\sum_{TB\text{-}nontreat4}$$

Overall TB case reduction: $\sum_{TB\text{-}CXR\text{-}based<35}-\sum_{TB\text{-}nontreat}$

Overall SAEs:

$$\sum_{SAE\text{-}CXR\text{-}based<35}=e*w1*(r1*St1+(1-r1)*S6h1)+e*w2*(r2*St2+(1-r2)*S6h2)$$

Overall acquired INH-R cases:

$$\sum_{INHR\text{-}CXR\text{-}based<35}=a*(e*w1*((1-r1)*n1*(n6h1*(1-S6h1)+S6h1))+e*w2*((1-r2)*n2*(n6h2*(1-S6h2)+S6h2))$$

(5) IPT for contacts under 19 years only (National TB Program in Peru), introduce parameters similar to Peruvian household contacts population: w2a for age15-19 subgroup, w2b for age20-34 subgroup , w2=w2a+w2b:

Overall TB cases:

$$\sum_{TB\text{-}IPT<19}=e*w1*\sum_{TB\text{-}IPT\text{-}all1}+e*w2a*\sum_{TB\text{-}IPT\text{-}all12}+e*w2b*\sum_{TB\text{-}nontreat2}+e*w3*\sum_{TB\text{-}nontreat3}+e*w4*\sum_{TB\text{-}nontreat4}$$

Overall TB case reduction: $\sum_{TB\text{-}IPT<19}-\sum_{TB\text{-}nontreat}$

Overall SAEs:

$$\sum_{SAE\text{-}IPT<19}=e*(w1*S6h1+w2a*S6h2)$$

Overall acquired INH-R cases:

$$\sum_{INHR\text{-}IPT<19}=a*(e*w1*\sum_{TB\text{-}IPT\text{-}all1}+e*w2a*\sum_{TB\text{-}IPT\text{-}all12})$$

(6) CXR-based regimen for contacts under 19 years only, introduce parameters similar to Peruvian household contacts population: w2a for age15-19 subgroup, w2b for age20-34 subgroup , w2=w2a+w2b:

Overall TB cases:

$$\sum_{TB\text{-}CXR\text{-}based<19}=e*w1*\sum_{TB\text{-}CXR\text{-}based1}+e*w2a*\sum_{TB\text{-}CXR\text{-}based2}+$$

© 2025 Tan Q et al. *JAMA Network Open*.

$$e*w2b*\sum_{TB\text{-}nontreat2}+e*w3*\sum_{TB\text{-}nontreat3}+e*w4*\sum_{TB\text{-}nontreat4}$$

Overall TB case reduction: $\sum_{TB\text{-}CXR\text{-}based<19}$—$\sum_{TB\text{-}nontreat}$

Overall SAEs:

$$\sum_{SAE\text{-}CXR\text{-}based<19}=e*w1*(r1*St1+(1-r1)*S6h1)+e*w2a*(r2*St2+(1-r2)*S6h2)$$

Overall acquired INH-R cases:

$$\sum_{INHR\text{-}CXR\text{-}based<19}=a*(e*w1*((1-r1)*n1*(n6h1*(1-S6h1)+S6h1))+e*w2a*((1-r2)*n2*(n6h2*(1-S6h2)+S6h2))$$

Scenario Analysis with varying uptake rates

We used the sample of the hypothetical HHC cohort to project the overall TB cases, overall TB case reduction, overall SAEs and overall cases with acquired isoniazid resistance despite IPT with each of the six-management strategy scenario, assuming e =1,000 across all age group in a HHC cohort, assuming uptake rate of IPT or active TB treatment the same across all age group:

K= uptake rate of IPT or active TB treatment

(0) Monitoring with non-treatment:

$$\sum_{TB\text{-}nontreat}=e*w1*(r1*p1+(1-r1)*n1)+e*w2*(r2*p2+(1-r2)*n2)+e*w3*(r3*p3+(1-r3)*n3)+ e*w4*(r4*p4+(1-r4)*n4)$$

(1) IPT for all contacts

Overall TB cases:

$\sum_{TB\text{-}IPT\text{-}ALLUPTAKE\%}=\sum_{TB\text{-}IPT\text{-}ALL}$ ***K**+$\sum_{TB\text{-}nontreat}$***(1-K)**

Overall TB case reduction= $\sum_{TB\text{-}IPT\text{-}ALLUPTAKE\%}$—$\sum_{TB\text{-}nontreat}$

Overall SAEs:

$\sum_{SAE\text{-}IPT\text{-}ALLUPTAKE\%}=\sum_{SAE\text{-}IPT\text{-}ALL}$***K**

Overall acquired INH-R cases:

$\sum_{INHR\text{-}IPT\text{-}ALLUPTAKE\%}=a*\sum_{TB\text{-}IPT\text{-}ALLUPTAKE\%}$ ***K**

© 2025 Tan Q et al. *JAMA Network Open*.

(2) CXR-based regimen for all contacts

Overall TB cases:

$\sum_{\text{TB-CXR-basedUPTAKE\%}}=\sum_{\text{TB-CXR-based}}*\mathbf{K}+\sum_{\text{TB-nontreat}}*\mathbf{(1-K)}$

Overall TB case reduction= $\sum_{\text{TB-CXR-baseduptake\%}}-\sum_{\text{TB-nontreat}}$

Overall SAEs:

$\sum_{\text{SAE-CXR-basedUPTAKE\%}}=\sum_{\text{SAE-CXR-based}}*\mathbf{K}$

Overall acquired INH-R cases:

$\sum_{\text{INHR-CXR-baseduptake\%}}=a*\sum_{\text{INHR-CXR-based}}*\mathbf{K}$

(3) IPT for contacts under 35 years only

Overall TB cases:

$\sum_{\text{TB-IPT<35UPTAKE\%}}=e*w1*\mathbf{K}*(r1*p1*p6h1+(1-r1)*n1*p6h1)+e*w2*\mathbf{K}*(r2*p2*p6h2+(1-r2)*n2*n6h2)+$
$e*w3*(r3*p3+(1-r3)*n3)+e*w4*(r4*p4+(1-r4)*n4)+$
$(e*w1*(r1*p1*t1+(1-r1)*n1*n6h1)+e*w2*(r2*p2*t2+(1-r2)*n2*n6h2)*\mathbf{(1-K)}$

Overall TB case reduction= $\sum_{\text{TB-IPT<35UPTAKE\%}}-\sum_{\text{TB-nontreat}}$

Overall SAEs:

$\sum_{\text{SAE-IPT<35UPTAKE\%}}=e*(w1*S6h1+w2*S6h2)*\mathbf{K}$

Overall acquired INH-R cases:

$\sum_{\text{INHR-IPT<35UPTAKE\%}}=a*\sum_{\text{TB-IPT<35}}*\mathbf{K}$

(4) CXR-based regimen for contacts under 35 years only

Overall TB cases:

$\sum_{\text{TB-CXR-based <35UPTAKE\%}}=e*w1*\mathbf{K}*(r1*p1*t1+(1-r1)*n1*n6h1)+e*w2*\mathbf{K}*(r2*p2*t2+(1-r2)*n2*n6h2)+$
$e*w3*(r3*p3+(1-r3)*n3)+e*w4*(r4*p4+(1-r4)*n4)$
$(e*w1*(r1*p1*t1+(1-r1)*n1*n6h1)+e*w2*(r2*p2*t2+(1-r2)*n2*n6h2)*\mathbf{(1-K)}$

Overall TB case reduction: $\sum_{\text{TB-CXR-based<35uptake\%}}-\sum_{\text{TB-nontreat}}$

© 2025 Tan Q et al. *JAMA Network Open*.

Overall SAEs:

$$\sum_{SAE\text{-}CXR\text{-}based<35UPTAKE\%}=(e*w1*(r1*St1+(1-r1)*S6h1)+e*w2*(r2*St2+(1-r2)*S6h2))*\mathbf{K}$$

Overall acquired INH-R cases:

$$\sum_{INHR\text{-}CXR\text{-}based<35UPTAKE\%}=a*(\ e*w1*((1-r1)*n1*n6h1)+e*w2*((1-r2)*n2*n6h2))*\mathbf{K}$$

(5) IPT for contacts under 19 years only (National TB Program in Peru), introduce parameters similar to Peruvian household contacts population: w2a for age15-19 subgroup, w2b for age20-34 subgroup , w2=w2a+w2b:

Overall TB cases:

$$\sum_{TB\text{-}IPT<19UPTAKE\%}=(e*w1*(r1*p1*p6h1+(1-r1)*n1*n6h1)+e*w2a*(r2*p2*p6h2+(1-r2)*n2*n6h2))*\mathbf{K}+ e*w2b*(r2*p2+(1-r2)*n2)+e*w3*(r3*p3+(1-r3)*n3)+e*w4*(r4*p4+(1-r4)*n4) +(e*w1*(r1*p1*t1+(1-r1)*n1*n6h1)+e*w2a*(r2*p2*t2+(1-r2)*n2*n6h2)*\mathbf{(1-K)}$$

Overall TB case reduction: $\sum_{TB\text{-}IPT<19UPTAKE\%}$—$\sum_{TB\text{-}nontreat}$

Overall SAEs:

$$\sum_{SAE\text{-}IPT<19UPTAKE\%}=e*(w1*S6h1+w2a*S6h2)*\mathbf{K}$$

Overall acquired INH-R cases:

$$\sum_{INHR\text{-}IPT<19uptake\%}=a*\mathbf{K}*(e*w1*(r1*p1*p6h1+(1-r1)*n1*n6h1)+e*w2a*(r2*p2*p6h2+(1-r2)*n2*n6h2))$$

(6) CXR-based regimen for contacts under 19 years only, introduce parameters similar to Peruvian household contacts population: w2a for age15-19 subgroup, w2b for age20-34 subgroup , w2=w2a+w2b:

Overall TB cases:

$$\sum_{TB\text{-}CXR\text{-}based<19uptake\%}=(e*w1*(r1*p1*t1+(1-r1)*n1*n6h1)+e*w2a*(r2*p2*t2+(1-r2)*n2*n6h2))*\mathbf{K}+ e*w2b*(r2*p2+(1-r2)*n2)+e*w3*(r3*p3+(1-r3)*n3)+e*w4*(r4*p4+(1-r4)*n4)+ +(e*w1*(r1*p1*t1+(1-r1)*n1*n6h1)+e*w2a*(r2*p2*t2+(1-r2)*n2*n6h2)*\mathbf{(1-K)}$$

Overall TB case reduction: $\sum_{TB\text{-}CXR\text{-}based<19UPTAKE\%}$—$\sum_{TB\text{-}nontreat}$

Overall SAEs:

$$\sum_{SAE\text{-}CXR\text{-}based<19UPTAKE\%}=(e*w1*(r1*St1+(1-r1)*S6h1)+e*w2a*(r2*St2+(1-r2)*S6h2))*\mathbf{K}$$

© 2025 Tan Q et al. *JAMA Network Open*.

Overall acquired INH-R cases:

$$\sum_{\text{INHR-CXR-based<19UPTAKE\%}}=a*(e*w1*((1-r1)*n1*n6h1)+e*w2a*((1-r2)*n2*n6h2))*\mathbf{K}$$

III. Details of parameter estimation

Effect of Standard First-Line Drug Regimens on Adult Contacts with Baseline Abnormal CXR

We assessed the efficacy of the standard 6-month regimen (2HRZE/4HR) recommended in the WHO 2022 guidelines for adult contacts aged over 15 years with baseline abnormal CXR Data were sourced from Study 31/A5349, a multicenter, phase 3 trial conducted by the CDC and NIH AIDS Clinical Trials Group (*Dorman et al., 2021*).[9] This trial included 2,343 participants aged ≥12 years with newly diagnosed pulmonary TB from 13 countries, and the primary efficacy outcome was survival free of TB at 12 months post-randomization.

Treatment Outcomes: The efficacy of the standard regimen was extracted from the study's intention-to-treat analysis. Among assessable participants, 656 of 726 (90.4%) had favorable outcomes, while unfavorable outcomes (9.6%) included culture positivity at the endpoint, TB recurrence, treatment discontinuation due to severe side effects, loss to follow-up, withdrawal, or death. We assumed that in the absence of treatment, 100% of individuals in the control group would remain diseased at the endpoint.

To estimate age-stratified efficacy, we utilized data from a related Indian trial (*Prajapati et al., 2023*)[10] which analyzed outcomes in 8,301 patients with drug-susceptible TB. The study, with a mean age of 35.3 years, reported 87.9% successful and 12.1% unsuccessful outcomes using a similar first-line fixed-dose combination regimen (2HRZE/4HRE). Although the continuation phase in *Prajapati et al.* differed slightly (4HRE vs. 4HR), the overall treatment efficacy aligned closely with that in Study 31/A5349, validating the use of its age-stratified data for weighting. We extracted risks of unsuccessful outcomes for three age groups from Prajapati et al.: Age 15–35: (164+193)/(1982+1700) = 0.097; Age 35–65: (115+143+109)/(1068+925+690) = 0.137; Age >65: 115/508 = 0.226

From Study 31/A5349, we estimated age-group population sizes assuming a normal distribution based on the median age (30.9 years; range: 13.7–77.5): Age 12–35: 498;Age 35–65: 261; Age >65: 9.

Using Prajapati et al.'s age-specific risks as weights, the adjusted age-group risks for Study 31/A5349 were calculated as follows:
Age group risk=Total unfavorable outcomes in Study 31×Age weight riskAge group risk=Total unfavorable outcomes in Study 31×Age weight risk
Age weight risk=Riskpraja (age group)∑(Riskpraja×Group size)Age weight risk=∑(Riskpraja ×Group size)Riskpraja (age group)

Risk of acquiring resistance when TB progresses from latent infection or early TB states despite preventive treatment

To estimate the probability of acquiring isoniazid resistance (INH-R) among individuals who develop TB after receiving isoniazid preventive therapy (IPT), we synthesized evidence from a published meta-analysis and national surveillance data(eTable2).[15] We assumed that the excess INH resistance attributable to IPT use could be estimated by multiplying the background prevalence of INH resistance among new TB cases by the excess relative risk:

© 2025 Tan Q et al. *JAMA Network Open*.

$P(\text{acquired INH-R})=(RR-1)\times P0P(\text{acquired INH-R})=(RR-1)\times P0$

where P0 represents the prevalence of isoniazid resistance in new TB cases among household contacts not receiving IPT. Based on the 2005–2006 National Surveillance Study in Peru,[17] we used a background INH-R prevalence of 6.3%. The point estimate of acquired resistance was therefore calculated as:

$(1.45-1)\times 0.063=0.028(2.8\%)(1.45-1)\times 0.063=0.028(2.8\%)$

To account for uncertainty in the relative risk (RR) estimate, we assumed that RR follows a log-normal distribution. We derived the distribution parameters from the reported point estimate (RR = 1.45) and 95% confidence interval (0.85–2.47) of the 2006 meta-analysis. Using these parameters, we performed 10,000 Monte Carlo simulations, drawing RR values from the log-normal distribution.

To capture the uncertainty in the derived probability of acquired resistance, we fit a Beta distribution to the 10,000 simulated probability values using the method of moments. The shape parameters α and β were estimated from the sample mean and variance. This Beta distribution was subsequently used in probabilistic sensitivity analyses. We report the mean and 95% uncertainty interval derived from the simulated values in Table 1 and the shape parameters in eTable 3.

VI. The model incorporated the age structure and CXR abnormality prevalence typical of a household contact cohort in Peru, as detailed in eTable S1.

© 2025 Tan Q et al. *JAMA Network Open*.

eTable 1. Parameters From the Real Peruvian Household Contact Cohort

Characteristics	N (%)
Total cohort participants, exposed to a culture-positive TB patient	12,767 (100)
Gender (Female %)	5,106 (40)
HHC cohort age structure (year), n=12,767	
0-14	4,212 (33)
15-18	1,012 (7.9)
19-34	3,346 (26)
35-64	3,573 (28)
Over 64	572 (4.5)
Total incident TB cases over one year follow-up	444 (3.4)
Baseline TST screening positive	5,313 (42)
Rate of IPT uptake among HHCs less than 19 years	2,571 /5,224 (49)
With both symptom and CXR screening and ruled of TB disease at baseline among baseline TST positive HHCs	2,540 /5,313 (49)
Asymptomatic status at baseline screening	2,105 /2,540 (83)
Baseline CXR abnormality prevalence in screened age groups (n=2,540)	
0-14	35 /793 (4)
15-34	42 /735 (6)
35-64	78 /907 (9)
Over65	14 /105 (14)
HIV-positivity among HHCs underwent CXR and symptom screening	4/ 2,540 (0.2)
Starting IPT among fully screened HHC aged 0-14 years	451 /793 (57)
Total incident TB cases among fully screened HHCs over one-year follow-up	87 /2540 (3.4)
Incident TB cases among CXR-abnormal HHCs over one-year follow-up	40 /170 (24)

© 2025 Tan Q et al. *JAMA Network Open*.

eResults.

When applying 4R across the six intervention scenarios targeting the entire household contact population, it was associated with a lower incidence of serious adverse events (SAEs)—6 (IQR 6–7) per 1,000 contacts treated(eTable 9)—compared to 3HP, which had a higher incidence of 21 (IQR 20–23) per 1,000 contacts treated under full population scale-up (eTable 10). Similarly, both rifamycin-based regimens showed increased SAE incidence in older adults relative to younger age groups; however, 4R consistently had the lowest SAE rates across all age groups compared to both 3HP and IPT(eTable 6, eFigure 6). When applying the three TPT regimens to the entire HHC population under intervention scenarios 1a and 2a, 4R was associated with the lowest incidence of SAEs, while 3HP had the highest. These results are illustrated in eFigure 7.Assuming that 4R and 3HP have similar efficacy to IPT for subclinical TB, the more favorable safety profile of 4R suggests it may be the preferred option—especially when scaling up preventive treatment strategies to the broader HHC population.

When the overall uptake rate for screening and IPT was reduced to 50% across the entire household contact population, the number of TB cases averted decreased from 25 (IQR: 10–15) to 13 (IQR: 10–15). The number of SAEs declined from 16 (IQR: 15–18) to 8 (IQR: 7–9), and cases of isoniazid resistance decreased from 0.61 (IQR: 0.21–0.82) to 0.3 (IQR: 0.1–0.4) per 1,000 contacts treated (eTable 8). As uptake rates for both IPT and active TB treatment declined from 100% to 75%, 50%, and 25%, the differences in TB case reduction and SAEs between the screening-and-IPT strategy and the screening-and-treatment strategy also became less pronounced across age groups per 1,000 contacts treated (eFigure 5).

© 2025 Tan Q et al. *JAMA Network Open*.

Supplemental Tables and Figures

eTable 2. Source Data Extracted for Each Parameter

Parameter	Intervention	Age group	Source	Point estimate	95% lower	95% upper	Note
Risk of incident disease among contacts with CXR-	None	00-14	Huang, et al 2022	0.046	0.023	0.069	Risk as probability at the end of one year follow-up, Point estimate as num/denom; CLs can be regenerated
Risk of incident disease among contacts with CXR-	None	15-34	Tan, et al 2024	0.020	0.010	0.031	Same as above
Risk of incident disease among contacts with CXR-	None	35-64	Tan, et al 2024	0.012	0.005	0.019	Same as above, because of small sample size in age over65, we combined age 35-65 group and age over65 group , pooled the probabilities by sample , estimate size risk= 11/(830+90)
Risk of incident disease among contacts with CXR-	None	65+	Tan, et al 2024	0.012	0.005	0.019	Same as above
Risk of incident disease among contacts with CXR+	None	00-14	Huang, et al 2022	0.588	0.354	0.822	Risk as probability at the end of one year follow-up, Point estimate by num/denom; CLs can be regenerated
Risk of incident disease among contacts with CXR+	None	15-34	Tan, et al 2024	0.405	0.256	0.553	Same as above
Risk of incident disease among contacts with CXR+	None	35-64	Tan, et al 2024	0.103	0.036	0.170	Same as above
Risk of incident disease among contacts with CXR+	None	65+	Tan, et al 2024	0.133	0.000	0.305	Same as above
Risk ratio with 6month IPT among contacts with CXR-	6-month Isoniazid (IPT)	00-14	Huang, et al 2022	0.307	0.124	0.763	Point estimate RISK as num/denom; CLs can be regenerated
Risk ratio with 6month IPT among contacts with CXR-	6-month Isoniazid (IPT)	15-34	Zinner et,al 2017, network metaanalysis	0.484	0.314	0.744	assume the effect is consistent across all adult age groups and for one year follow-up , we estimated pooled RR using data of three trials on 6H compared no Tx extracted from network meta-analysis study
Risk ratio with 6month IPT among contacts with CXR-	6-month Isoniazid (IPT)	35-64	Zinner et,al 2017, network metaanalysis	0.484	0.314	0.744	same as above

© 2025 Tan Q et al. *JAMA Network Open*.

Parameter	Intervention	Age group	Source	Point estimate	95% lower	95% upper	Note
Risk ratio with 6month IPT among contacts with CXR-	6-month Isoniazid (IPT)	65+	Zinner et,al 2017, network metaanalysis	0.484	0.314	0.744	same as above
Risk ratio with 6month IPT among contacts with CXR+	6-month Isoniazid (IPT)	00-14	Huang, et al 2022	0.283	0.059	1.364	Point estimate RISK as num/denom; CLs can be regenerated
Risk ratio with 6month IPT among contacts with CXR+	6-month Isoniazid (IPT)	15-34	Gray et al 2023 metaanalysis	0.63	0.35	1.13	assume the effect is consistent across all adult groups over 15 years and for one year follow-up , we estimated pooled RR by using random-effects meta-analysis approach. (R package (meta))
Risk ratio with IPT among contacts with CXR+	6-month Isoniazid (IPT)	35-64	Gray et al 2023 metaanalysis	0.63	0.35	1.13	same as above
Risk ratio with IPT among contacts with CXR+	6-month Isoniazid (IPT)	65+	Gray et al 2023 metaanalysis	0.63	0.35	1.13	same as above
Risk ratio with curative TB treatment among contacts with CXR+	2HRZE2HR (4month)	00-14	Turkova,et al 2022 NEJM,SHINE trial	0.030	0.017	0.045	Point estimate RISK as num/denom; risk of control group=100%; Point estimate RR = RISK of intervention/RISK of control ,CLs can be regenerated
Risk ratio with curative TB treatment among contacts with CXR+	2HRZE4HR(6month)	15-34	Dorman,et al 2021,NEJMThe Tuberculosis Trials Consortium (Study 31/A5349); Prajapati, et al2023	0.079	0.0549	0.0102	Point estimate risk in intervention group as num of total cases multiply age weight risk; risk of control group =100%. Point estimate RR =RISK of intervention/RISK of control =0.079 ,CLs can be regenerated by using point estimate RR and the denom age population size n=498
Risk ratio with curative TB treatment among contacts with CXR+	2HRZE4HR(6month)	35-64	Dorman,et al 2021,NEJMThe Tuberculosis Trials Consortium (Study 31/A5349;	0.111	0.073	0.149	Point estimate risk in intervention group as num of total cases multiply age weight risk; risk of control group =100%. Point estimate RR =RISK of intervention/RISK of control=0.111 ,CLs can be regenerated by using point estimate RR and the denom age population size n=261

© 2025 Tan Q et al. *JAMA Network Open*.

			Prajapati, et al2023				

Parameter	Intervention	Age group	Source	Point estimate	95% lower	95% upper	Note
Risk ratio with curative TB treatment among contacts with CXR+	2HRZE4HR(6month)	65+	Dorman,et al 2021,NEJMThe Tuberculosis Trials Consortium (Study 31/A5349; Prajapati, et al2023	0.183	0	0.43	Point estimate risk in intervention group as num of total cases multiply age weight risk; risk of control group =100%. Point estimate RR =RISK of intervention/RISK of control =0.183,CLs can be regenerated by using point estimate RR and the denom age population size n=9
Risk of SAE among contacts with CXR-	6-month Isoniazid (IPT)	00-14	Menzies, et al.2018	0.002	0	0.007	Point estimate as num/denom, CLs can be regenerated
Risk of SAE among contacts with CXR-	6-month Isoniazid (IPT)	15-34	Campbell, et al.2020	0.015	0.010	0.023	age stratified risk data and 95 CIs directly provided by authors of source paper(Menzies et al 2018) , which is published on the modeling paper (Courtney et al , 2020) as supplemental data
Risk of SAE among contacts with CXR-	6-month Isoniazid (IPT)	35-64	Campbell, et al.2020	0.028	0.021	0.037	same above
Risk of SAE among contacts with CXR-	6-month Isoniazid (IPT)	65+	Campbell, et al.2020	0.055	0.027	0.109	same above
Risk of SAE among contacts with abnormal CXR	2HRZE/2HR	00-14	Turkova,et al 2022 NEJM,SHINE trial	0.078	0.057	0.099	Point estimate RISK as num/denom; CLs can be regenerated
Risk of SAE among contacts with abnormal CXR	2HRZE/4HR	15-34	Yee et al 2003	0.054	0.024	0.084	estimate risk of incident rate by extracting total incident rate , age group population and weight by adjust HR, 95CI can be regenerated
Risk of SAE among contacts with abnormal CXR	2HRZE/4HR	35-64	Yee et al 2003	0.093	0.043	0.207	estimate risk of incident rate by extracting total incident rate , age group population and weight by adjust HR
Risk of SAE among contacts with abnormal CXR	2HRZE/4HR	65+	Yee et al 2003	0.157	0.071	0.343	estimate risk of incident rate by extracting total incident rate , age group population and weight by adjust HR
Risk ratio of INH resistance acquisition in TB cases despite IPT	6-month Isoniazid (IPT)	All	Balcells et al., 2016	1.45	0.85	2.47	conservatively estimate the risk of acquiring isoniazid monoresistance following unsuccessful IPT in pan-susceptible TB-exposed

© 2025 Tan Q et al. *JAMA Network Open*.

							individuals who are initially TST-positive
Prevalence of INH-resistance in Peru among newly diagnosed TB patients	6-month Isoniazid (IPT)	All	Asencios L,et al 2009	6.3%	NA	NA	National Surveillance Study in Peru (2005–2006), Hr-TB without MDR-TB rate in new cases was 6.3%
Risk of SAE among contacts with CXR- or CXR+	3HP	00-14	Melnychuk,et al 2023	0.005	0	0.011	Point estimate RISK as num/denom using unpublished age- stratified data provided by the author of the study of "a systematic Review and Meta-Analysis of Tuberculous Preventative Therapy Adverse Events" (by Melnychuk et,al, 2023)
Risk of SAE among contacts with CXR- or CXR+	3HP	15-34	same above	0.019	0.013	0.026	same above
Risk of SAE among contacts with CXR- or CXR+	3HP	35-64	same above	0.037	0.029	0.045	same above
Risk of SAE among contacts with CXR- or CXR+	3HP	65+	same above	0.064	0.026	0.103	same above
Risk of SAE among contacts with CXR- or CXR+	4R	00-14	same above	0	0	0	same above
Risk of SAE among contacts with CXR- or CXR+	4R	15-34	same above	0.009	0.004	0.014	same above
Risk of SAE among contacts with CXR- or CXR+	4R	35-64	same above	0.009	0.004	0.013	same above
Risk of SAE among contacts with CXR- or CXR+	4R	65+	same above	0.02	0	0.046	same above

TST+= Tuberculin Skin Test positive. CXR+= Abnormal chest X-ray screening result. CXR-= Normal chest X-ray screening result. 2HRZE/4HR=2 months of HRZE followed by 4 months of HR. 2HRZE/2HR= 2 months of HRZE followed by 2 months of HR. 6H= Six-month daily isoniazid preventive therapy(IPT).
H= Isoniazid. R= Rifampin. Z= Pyrazinamide. E= Ethambutol
SAE= Severe Adverse Event.

© 2025 Tan Q et al. *JAMA Network Open*.

eTable 3. Distributional Assumptions for Model Parameters

B = beta distribution; LN = log-normal distribution; E=gamma distribution

Parameter description by CXR results	Intervention	Age Group (years)	Distribution
Progression risk in CXR-	None	00-14	B(14.6135456,303.072228313043)
Progression risk in CXR-	None	15-34	B(13.6390222222222,668.312088888889)
Progression risk in CXR-	None	35-64	B(11.305058846479,923.931627544054)
Progression risk in CXR-	None	65+	B(11.305058846479,923.931627544054)
Progression risk in CXR+	None	00-14	B(9.41212931427136,6.58849051998995)
Progression risk in CXR+	None	15-34	B(16.583510476951,24.387515407281)
Progression risk in CXR+	None	35-64	B(7.91646466164524,69.2690657893958)
Progression risk in CXR+	None	65+	B(2.41175008709327,15.6763755661063)
Risk ratio with treatment in CXR+	6H	00-14	LN(-1.69665810700598,0.932040477304587)
Risk ratio with treatment in CXR+	6H	15-34	LN(-2.01071398842326,0.0399627174259857)
Risk ratio with treatment in CXR+	6H	35-64	LN(-2.01071398842326,0.0399627174259857)
Risk ratio with treatment in CXR+	6H	65+	LN(-2.01071398842326,0.0399627174259857)
Risk ratio with treatment in CXR-	6H	00-14	LN(-1.30446081630318,0.498140977078668)
Risk ratio with treatment in CXR-	6H	15-34	LN(-0.751548497383503,0.223836266686181)
Risk ratio with treatment in CXR-	6H	35-64	LN(-0.751548497383503,0.223836266686181)
Risk ratio with treatment in CXR-	6H	65+	LN(-0.751548497383503,0.223836266686181)
Risk ratio with treatment in CXR+	2HRZE2HR (4month)	00-14	LN(-3.53412827665938,0.234820694741328)
Risk ratio with treatment in CXR+	2HRZE4HR(6month)	15-34	LN(-2.55462785064686,0.144438494300015)
Risk ratio with treatment in CXR+	2HRZE4HR(6month)	35-64	LN(-2.21512994036043,0.173665809379506)
Risk ratio with treatment in CXR+	2HRZE4HR(6month)	65+	LN(-1.84922935935387,0.55309856051699)
SAE probability in CXR+	6H	00-14	B(1.15958861370014,578.634718236369)
SAE probability in CXR+	6H	15-34	B(21.2627981971801,1357.9196121381)
SAE probability in CXR+	6H	35-64	B(45.1939550148373,1568.03374355827)

© 2025 Tan Q et al. *JAMA Network Open*.

Parameter description by CXR results	Intervention	Age Group (years)	Distribution
SAE probability in CXR+	6H	65+	B(6.44144817333538,110.424825828607)
SAE probability in CXR-	6H	00-14	B(1.15958861370014,578.634718236369)
SAE probability in CXR-	6H	15-34	B(21.2627981971801,1357.9196121381)
SAE probability in CXR-	6H	35-64	B(45.1939550148373,1568.03374355827)
SAE probability in CXR-	6H	65+	B(6.44144817333538,110.424825828607)
SAE probability in CXR+	2HRZE2HR (4month)	00-14	B(48.8741904725969,577.131398133857)
SAE probability in CXR+	2HRZE4HR(6month)	15-34	B(11.720657664,205.328558336)
SAE probability in CXR+	2HRZE4HR(6month)	35-64	B(4.38885314527067,42.8031161587151)
SAE probability in CXR+	2HRZE4HR(6month)	65+	B(4.15879895389273,22.3303663575259)
Risk of mono-isoniazid-resistance acquisition among TB cases despite IPT	6H	All	B(1.41062156337131,42.4387668906104)
SAE probability in CXR+ or CXR-	3HP	00-14	E(3.17475935594556,634.951871189112)
SAE probability in CXR+ or CXR-	3HP	15-34	B(32.1814275881657,1661.5779191574)
SAE probability in CXR+ or CXR-	3HP	35-64	B(79.096778675,2058.653996325)
SAE probability in CXR+ or CXR-	3HP	65+	B(9.87234083966942,144.382984780165)
SAE probability in CXR+ or CXR-	4R	00-14	NA
SAE probability in CXR+ or CXR-	4R	15-34	B(12.325762944,1357.203453056)
SAE probability in CXR+ or CXR-	4R	35-64	B(15.2191024,1675.79227537778)
SAE probability in CXR+ or CXR-	4R	65+	E(2.90469476044924,145.234738022462)

TST+= Tuberculin Skin Test positive；CXR+= Abnormal chest X-ray screening result；CXR-= Normal chest X-ray screening result; 2HRZE/4HR=2 months of HRZE followed by 4 months of HR;2HRZE/2HR= 2 months of HRZE followed by 2 months of HR;6H= Six-month daily isoniazid preventive therapy(IPT); 4R= 4 months of daily rifampicin; 3HP= 3 months of weekly isoniazid and rifapentine H=Isoniazid; R= Rifampin; Z= Pyrazinamide; E= Ethambutol; P= rifapentine; SAE= Severe Adverse Event; B = beta distribution; LN = log-normal distribution; E=gamma distribution

© 2025 Tan Q et al. *JAMA Network Open*.

eTable 4. One-Year Risk of TB and SAEs by Age, Regimen, and Initial CXR Results

Age group (Years)	Treatment option	CXR-normal population Median (IQR) , per1,000 contacts		CXR-abnormal population Median (IQR) , per1,000 contacts	
		TB disease cases	SAE cases	TB disease cases	SAE cases
0-14	No Pharmacological Intervention	46.01(37.76-53.39)	NA	588.60(505.35-673.06)	NA
	6H	14.14(8.2-17.71)	2(1.64-2.32)	166.42 (55.68-200.86)	2(1.64-2.32)
	4R	14.14(8.2-17.71)	0(0-0)	166.42 (55.68-200.86)	0(0-0)
	3HP	14.14(8.2-17.71)	5.01 (2.93-6.54)	166.42 (55.68-200.86)	5.01 (2.93-6.54)
	TBD regimen	NA	NA	17.66(13.67-20.91)	78.1(70.71-85.23)
15-34	No Pharmacological Intervention	20.0(16.22-23.33)	NA	404.46(352.27-456.9)	NA
	6H	9.66(7.2-11.55)	15.41(13.1-17.52)	254.54(187.13-304.84)	15.42(13.1-17.52)
	4R	9.66(7.2-11.55)	8.98(7.15-10.58)	254.54(187.13-304.84)	8.98(7.15-10.58)
	3HP	9.66(7.2-11.55)	19.04(16.76-21.19)	254.54(187.13-304.84)	19.04(16.76-21.19)
	TBD regimen	NA	NA	31.75(26.37-36.48)	54.02(43.18-63.5)
35-64	No Pharmacological Intervention	12.1(9.55-14.3)	0(0-0)	102.70(78.02-123.88)	NA
	6H	5.85(4.27-7.08)	28.03(25.18-30.74)	64.57 (43.03-79.62)	28.03(25.18-30.74)
	4R	5.85(4.27-7.08)	8.98(7.15-10.58)	64.57 (43.03-79.62)	8.98(7.15-10.58)
	3HP	5.85(4.27-7.08)	37.05(34.18-39.75)	64.57 (43.03-79.62)	37.05(34.18-39.75)
	TBD regimen	NA	NA	11.36(8.25-13.79)	92.98(62.29-117.9)
over65	No Pharmacological Intervention	12.1(9.55-14.3)	0(0-0)	133.54(74.91-178.34)	NA
	6H	5.84 (4.27-7.08)	55.14(39.98-67.79)	84.26 (42.36-111)	55.14(39.98-67.79)
	4R	5.84 (4.27-7.08)	20.06(11.37-26.29)	84.26 (42.36-111)	20.06(11.37-26.29)
	3HP	5.84 (4.27-7.08)	63.98(49.87 to 76.01)	84.26 (42.36-111)	63.98(49.87 to 76.01)
	TBD regimen	NA	NA	24.56(10.04-31.66)	157.24(106.09-199.73)

SAEs= Severe Adverse Events ; CXR=Chest X-Ray; IQR= Interquartile range; 6H=6months of daily isoniazid; 4R=4 months of daily rifampicin; 3HP=3 months of weekly isoniazid and rifapentine

© 2025 Tan Q et al. *JAMA Network Open*.

eTable 5. Estimated Outcomes and Differences Between 2 Contact Management Strategies by Age Group

Age group (years)[a]	Intervention Strategy	Expected Case number			Differences in CXR-based treatment *vs.* universal IPT		
		TB Case Reduction	SAEs	INH-Resistance Acquisition	TB Cases	SAEs	INH-Resistance Acquisition
		Median (IQR) , per 1,000 contacts of age group					
0-14	Strategy1 (universal IPT)	-47.39(-57.79 to -39.35)	2.02 (0.67 to 2.78)	0.65(0.18 to 0.81)	NA	NA	NA
	Strategy2 (CXR-guidance)	-53.33(-60.74 to -45.84)	5.07(3.78 to 5.85)	0.46(0.14 to 0.6)	-5.94(-7.2 to -1.66)	2.51(2.2 to 2.8)	-0.19(-0.31 to -0.03)
15-34							
	Strategy1 (universal IPT)	-18.76(-23.77 to -14.12)	15.42(13.1 to 17.52)	0.79(0.29 to 1.08)	NA	NA	NA
	Strategy2 (CXR-guidance)	-31.59(-35.23 to -27.85)	17.73(15.45 to 19.85)	0.37(0.13 to 0.5)	-12.84(-15.55 to -9.18)	2.31(1.65 to 2.9)	-0.42(-0.57 to -0.15)
36-64							
	Strategy1 (universal IPT)	-8.87(-11.22 to -6.45)	28.03(25.18 to 30.74)	0.37(0.13 to 0.49)	NA	NA	NA
	Strategy2 (CXR-guidance)	-13.52(-15.77 to -10.99)	33.88(30.14 to 37.24)	0.22(0.08 to 0.29)	-4.65(-5.77 to -3.03)	5.84 (3.09 to 8.08)	-0.15(-0.2 to -0.05)
Over 65							
	Strategy1 (universal IPT)	-11.61(-15.19 to -7.34)	55.14(39.98 to 67.79)	0.56(0.18 to 0.75)	NA	NA	NA
	Strategy2 (CXR-guidance)	-19.51(-24.56 to -12.75)	69.44(54.52 to 82.35)	0.31(0.1 to 0.41)	-7.9(-10.49 to -4.21)	14.29(7 to 20.37)	-0.25(-0.34 to -0.07)

[a]CXR abnormality prevalence among household contacts free of TB disease based on EPI cohort of our previous study[4,5]: age 0-14 years: 4%; age 15-34 years: 6%; age 35-64 years: 9%,age >=65 years: 14%

IPT: Six-month daily isoniazid preventive therapy.
CXR: chest X-ray
IQR: Interquartile range

© 2025 Tan Q et al. *JAMA Network Open*.

eTable 6. Estimated Outcomes and Differences Between 2 Intervention Strategies by Age Group Applying 3HP or 4R

Age group (years)[a]	Intervention Strategy	3HP		4R	
		TB Case Reduction	SAEs	TB Case Reduction	SAEs
		Median (IQR) , per 1,000 contacts of age group			
0-14	Strategy1 (universal TPT)	-47.49(-57.95 to -39.45)	5.03(3 to 6.57)	-47.49(-57.95 to -39.45)	0
	Strategy2 (CXR-guidance)	-53.44(-60.85 to -45.92)	7.94(5.99 to 9.4)	-53.44(-60.85 to -45.92)	3.11(2.82 to 3.39)
15-34					
	Strategy1 (universal TPT)	-18.72(-23.79 to -14.04)	19.03(16.71 to 21.15)	-18.72(-23.79 to -14.04)	8.99(7.17 to 10.58)
	Strategy2 (CXR-guidance)	-32.09(-35.79 to-28.29)	21.13(18.84 to 23.2)	-32.09(-35.79 to-28.29)	11.69(9.88 to 13.31)
36-64					
	Strategy1 (universal TPT)	-9.12(-11.55 to -6.63)	37.02(34.22 to 39.72)	-9.12(-11.55 to -6.63)	8.95(7.31 to 10.36)
	Strategy2 (CXR-guidance)	-13.91(-16.2 to -11.3)	42.12(38.38 to 45.49)	-13.91(-16.2 to -11.3)	16.58(13.5 to 19.21)
Over 65					
	Strategy1 (universal TPT)	-12.28(-16.04 to -7.74)	64.35(50.16 to 76.63)	-12.28(-16.04 to -7.74)	19.22(11.03 to 25.11)
	Strategy2 (CXR-guidance)	-20.64(-25.98 to -13.56)	77.36(63.21 to 89.53)	-20.64(-25.98 to -13.56)	38.54(28.95 to 46.68)

[a]CXR abnormality prevalence among household contacts free of TB disease based on EPI cohort of our previous study[4,5]: age 0-14 years: 4%; age 15-34 years: 6%; age 35-64 years: 9%,age >=65 years: 14%

4R：4 months of daily rifampicin
3HP：3 months of weekly isoniazid and rifapentine

CXR: chest X-ray
IQR: Interquartile range

© 2025 Tan Q et al. *JAMA Network Open*.

**eTable 7.** Outcomes per 1000 Contacts by Intervention Scenarios Using 6H as the TPT Regimen					
**Intervention Scenario** [a]	**Expected number of TB cases, Median (IQR)**	**Expected reduction of TB cases vs. Non-intervention, Median (IQR)**	**Percentage of expected absolute TB case reduction, Median (IQR), %**[b]	**Expected number of SAE cases, Median (IQR)**	**Expected number of acquired mono-isoniazid resistance cases, Median (IQR)**
Monitoring without Pharmacological Intervention	44.02(40.41 to 47.33)	NA	NA	NA	NA
Scenario 1a (for all)	19.09(13.41 to 22.65)	-24.93(-30.53 to -20.65)	57(49 to 69)	16.25(14.85 to 17.57)	0.61(0.21 to 0.82)
Scenario 2a (for all)	10.98(8.11 to 12.93)	-33.04(-36.52 to -29.73)	75 (71 to 81)	20.32(18.78 to 21.77)	0.3(0.11 to 0.41)
Scenario 1b (for age<35 yrs only)	22.09(17.08 to 25.19)	-21.93(-26.84 to -18.15)	50 (43 to 60)	5.93(5.03 to 6.75)	0.49(0.16 to 0.64)
Scenario 2b (for age<35 yrs only)	15.64(12.92 to 17.6)	-28.38(-31.49 to -25.34)	64 (61 to 70)	8.22(7.33 to 9.02)	0.24(0.08 to 0.32)
Scenario 1c (for age<19 yrs only)(Status quo in Peru)	26.92(22.9 to 29.55)	-17.1(-20.86 to -14.24)	39 (34-47)	1.89(1.42 to 2.19)	0.28(0.08 to 0.36)
Scenario 2c (for age<19 yrs only)	23.92(21.21 to 26.13)	--20.1(-22.67 to -17.55)	46 (42-50)	3.19(2.73 to 3.52)	0.17(0.05 to 0.22)

[a] Applying parameters from a high-burden country similar to Peru: Prevalence of CXR abnormalities among household contacts without TB disease is as follows: age 0–14 years: 4%; age 15–34 years: 6%; age 35–64 years: 9%; age ≥65 years: 14%; Age structure of a Peruvian household population[9,11]; 0-14 years: 33;15-34 year:34%; 35-64 years: 28%; over 65:4.5% ; 15-19 years: 8%, 20-34 years: 26%.

[b] Percentage calculation: numerator= expected reduction of TB cases; denominator= expected TB cases in a nonpharmacological intervention scenario

IQR=Interquartile range. NA= not applicable. IPT=isoniazid preventive therapy. CXR= chest-X ray. HHCs= household contacts

© 2025 Tan Q et al. *JAMA Network Open*.

**eTable 8.** Outcomes per 1000 Contacts by Intervention Scenarios Using 6H as the TPT Regimen When Overall Uptake Rate Is 50%					
**Intervention Scenario [a]**	**Expected number of TB cases, Median (IQR)**	**Expected reduction of TB cases vs. Non-intervention, Median (IQR)**	**Percentage of expected absolute TB case reduction, Median (IQR), %[b]**	**Expected number of SAE cases, Median (IQR)**	**Expected number of acquired mono-isoniazid resistance cases, Median (IQR)**
Monitoring without Pharmacological Intervention	44.02(40.41 to 47.33)	NA	NA	NA	NA
Scenario 1a (for all)	31.43(27.69 to 34.33)	-12.58(-15.42 to -10.42)	28(24 to 35)	8.12(7.43 to 8.79)	0.3(0.1 to 0.41)
Scenario 2a (for all)	27,31(24.61 to 29.63)	-16.7(-18.46 to -15.03)	38 (36 to 41)	10.16(9.39 to 10.88)	0.15(0.06 to 0.21)
Scenario 1b (for age<35 yrs only)	32.99(29.46 to 35.75)	-11.03(-13.49 to -9.14)	25 (22 to 30)	2.97(2.51 to 3.38)	0.25(0.08 to 0.32)
Scenario 2b (for age<35 yrs only)	29.72(26.98 to 32.12)	-14.29(-15.86 to -12.76)	32 (30 to 35)	4.11(3.66 to 4.51)	0.12(0.04 to 0.16)
Scenario 1c (for age<19 yrs only)(Status quo in Peru)	35.44(32.15 to 38.17)	-8.58(-10.46 to -7.15)	19 (17-24)	0.95(0.71 to 1.1)	0.14(0.04 to 0.18)
Scenario 2c (for age<19 yrs only)	33.93(31.03 to 36.50)	-10.09(-11.37 to -8.81)	23 (21-25)	1.6(1.36 to 1.76)	0.09(0.03 to 0.11)

[a]CXR abnormality prevalence among household contacts free of TB disease based on EPI cohort of our previous study[4,5]: age 0-14 years: 4%; age 15-34 years: 6%; age 35-64 years: 9%,age >=65 years: 14%

IPT: Six-month daily isoniazid preventive therapy.
IQR: Interquartile range

© 2025 Tan Q et al. *JAMA Network Open*.

**eTable 9.** Outcomes per 1000 Contacts Under Intervention Scenarios Using 4R as the TPT Regimen				
**Intervention Scenario [a]**	**Expected number of TB cases, Median (IQR)**	**Expected reduction of TB cases vs. Non-intervention, Median (IQR)**	**Percentage of expected absolute TB case reduction, Median (IQR), %[b]**	**Expected number of SAE cases, Median (IQR)**
Monitoring without Pharmacological Intervention	44.02(40.41 to 47.33)	NA	NA	NA
Scenario 1a (for all)	18.86(13.13 to 22.46)	-25.16(-30.83 to -20.85)	28(24 to 35)	6.44(5.6 to 7.2)
Scenario 2a (for all)	10.62(7.73 to 12.57)	-33.4(-36.92 to -30.07)	38 (36 to 41)	11.38(10.27 to 12.41)
Scenario 1b (for age<35 yrs only)	21.96(16.92 to 25.07)	-22.06(-26.99 to -18.27)	25 (22 to 30)	3.07(2.45 to 3.61)
Scenario 2b (for age<35 yrs only)	15.43(12.72 to 17.39)	-28.58(-31.72 to -25.52)	32 (30 to 35)	5.02(4.38 to 5.57)
Scenario 1c (for age<19 yrs only)(Status quo in Peru)	26.87(22.83 to 29.49)	-17.15(-20.93 to -14.3)	19 (17-24)	0.71(0.57 to 0.84)
Scenario 2c (for age<19 yrs only)	23.84(21.14 to 26.05)	-20.17(-22.74 to -17.62)	23 (21-25)	2.07(1.88 to 2.24)

[a]CXR abnormality prevalence among household contacts free of TB disease based on EPI cohort of our previous study[4,5]: age 0-14 years: 4%; age 15-34 years: 6%; age 35-64 years: 9%,age >=65 years: 14%

4R：4 months of daily rifampicin

3HP：3 months of weekly isoniazid and rifapentine

CXR: chest X-ray

IQR: Interquartile range

© 2025 Tan Q et al. *JAMA Network Open*.

eTable 10. Outcomes per 1000 Contacts Under Intervention Scenarios Using 3HP as the TPT Regimen

**Intervention Scenario [a]**	**Expected number of TB cases, Median (IQR)**	**Expected reduction of TB cases vs. Non-intervention, Median (IQR)**	**Percentage of expected absolute TB case reduction, Median (IQR), %[b]**	**Expected number of SAE cases, Median (IQR)**
Monitoring without Pharmacological Intervention	44.02(40.41 to 47.33)	NA	NA	NA
Scenario 1a (for all)	18.86(13.13 to 22.46)	-25.16(-30.83 to -20.85)	28(24 to 35)	21.4(19.95 to 22.77)
Scenario 2a (for all)	10.62(7.73 to 12.57)	-33.4(-36.92 to -30.07)	38 (36 to 41)	25.09(23.54 to 26.54)"
Scenario 1b (for age<35 yrs only)	21.96(16.92 to 25.07)	-22.06(-26.99 to -18.27)	25 (22 to 30)	8.16(7.14 to 9.05)
Scenario 2b (for age<35 yrs only)	15.43(12.72 to 17.39)	-28.58(-31.72 to -25.52)	32 (30 to 35)	9.83(8.84 to 10.71)
Scenario 1c (for age<19 yrs only)(Status quo in Peru)	26.87(22.83 to 29.49)	-17.15(-20.93 to -14.3)	19 (17-24)	3.17(2.49 to 3.68)
Scenario 2c (for age<19 yrs only)	23.84(21.14 to 26.05)	-20.17(-22.74 to -17.62)	23 (21-25)	4.41(3.74 to 4.91)

[a]CXR abnormality prevalence among household contacts free of TB disease based on EPI cohort of our previous study[4,5]: age 0-14 years: 4%; age 15-34 years: 6%; age 35-64 years: 9%,age >=65 years: 14%

4R：4 months of daily rifampicin

3HP：3 months of weekly isoniazid and rifapentine

CXR: chest X-ray

IQR: Interquartile range

© 2025 Tan Q et al. *JAMA Network Open*.

eFigure 1. Plots of Probability Distribution Functions for Model Parameters in eTable 2

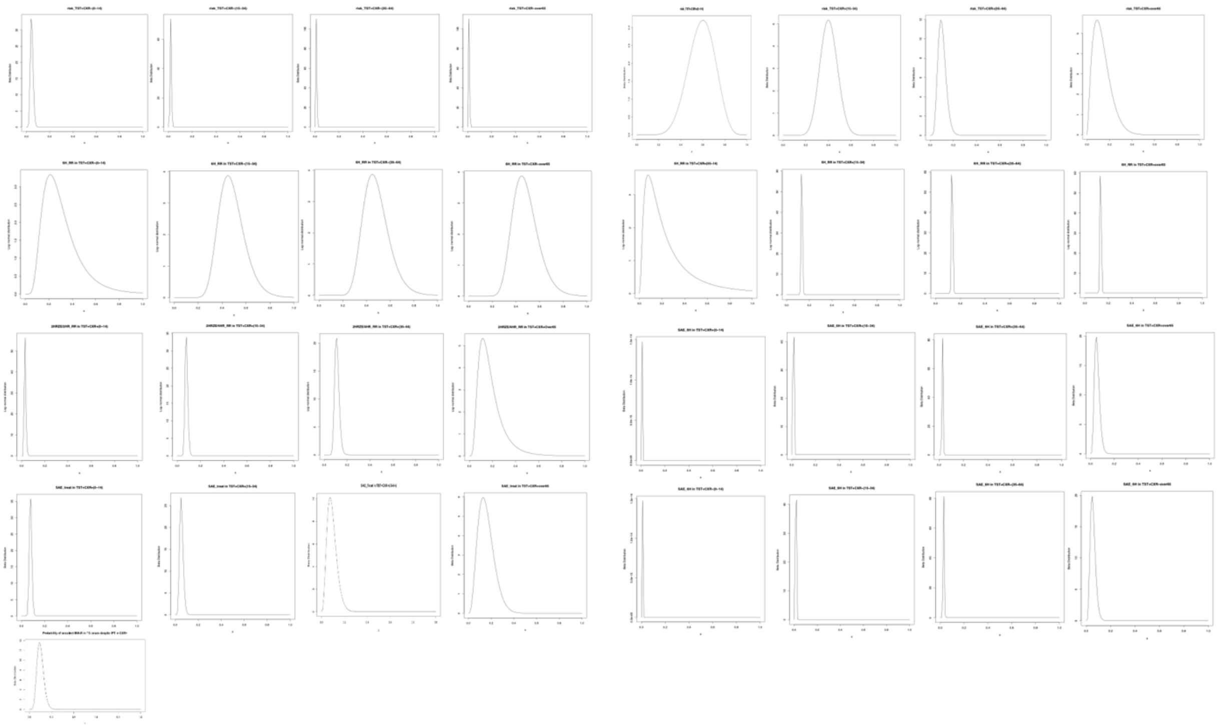

© 2025 Tan Q et al. *JAMA Network Open*.

eFigure 2. Outcomes of 2 Pharmacological Strategies Across Age Groups Using Different TPT Regimens (6H, 4R, and 3HP)

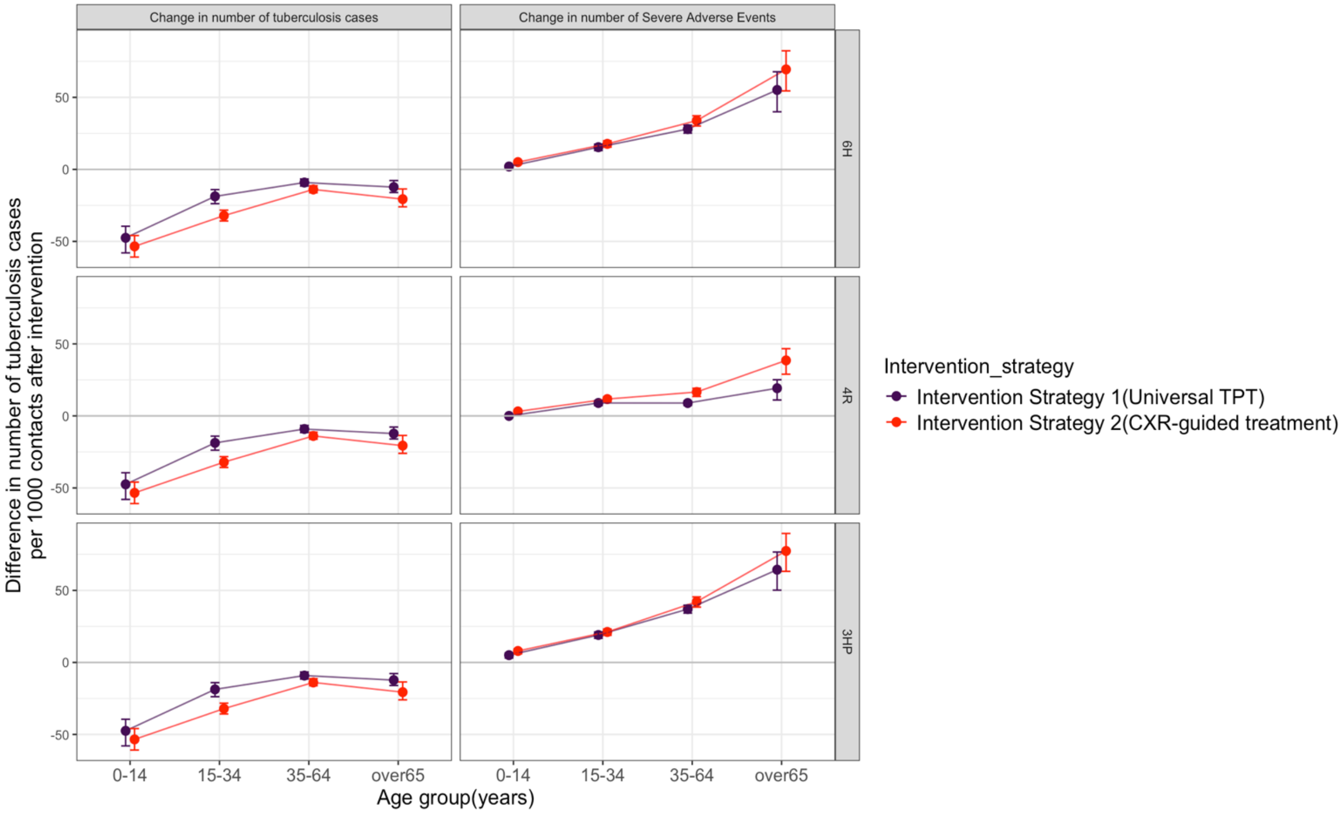

6H: 6 months of daily isoniazid
4R: 4 months of daily rifampicin
3HP: 3 months of weekly isoniazid and rifapentine

© 2025 Tan Q et al. *JAMA Network Open*.

eFigure 3. Comparison of Isoniazid Resistance Acquisition Cases Between 2 Strategies by Age Group

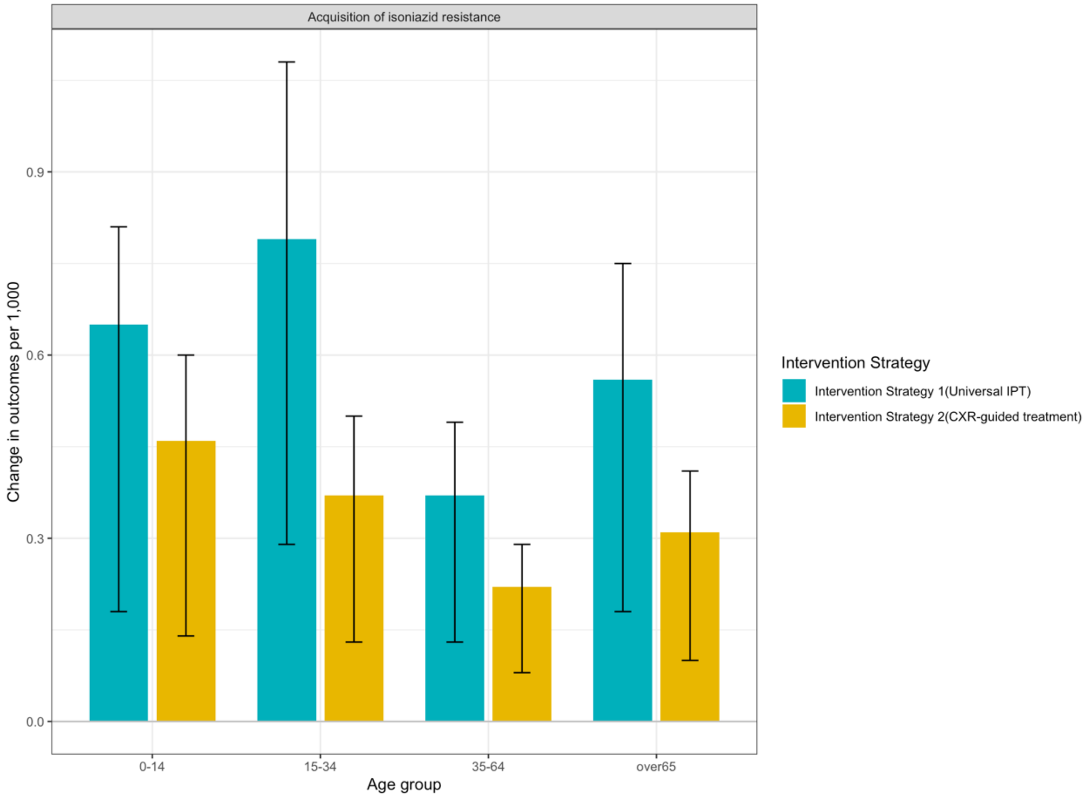

CXR abnormality prevalence among household contacts free of TB disease based on EPI cohort of our previous study[4,5]: age 0-14 years: 4%; age 15-34 years: 6%; age 35-64 years: 9%,age >=65 years: 14%

Error bars represent interquartile ranges.

IPT= Six-month daily isoniazid preventive therapy.
CXR= chest X-ray
H= Isoniazid
R= Rifampin
Z=Pyrazinamide
E=Ethambutol

© 2025 Tan Q et al. *JAMA Network Open*.

eFigure 4. Estimated Efficacy of 2 Strategies by 6 Intervention Scenarios Compared With Nonpharmacological Intervention Scenario

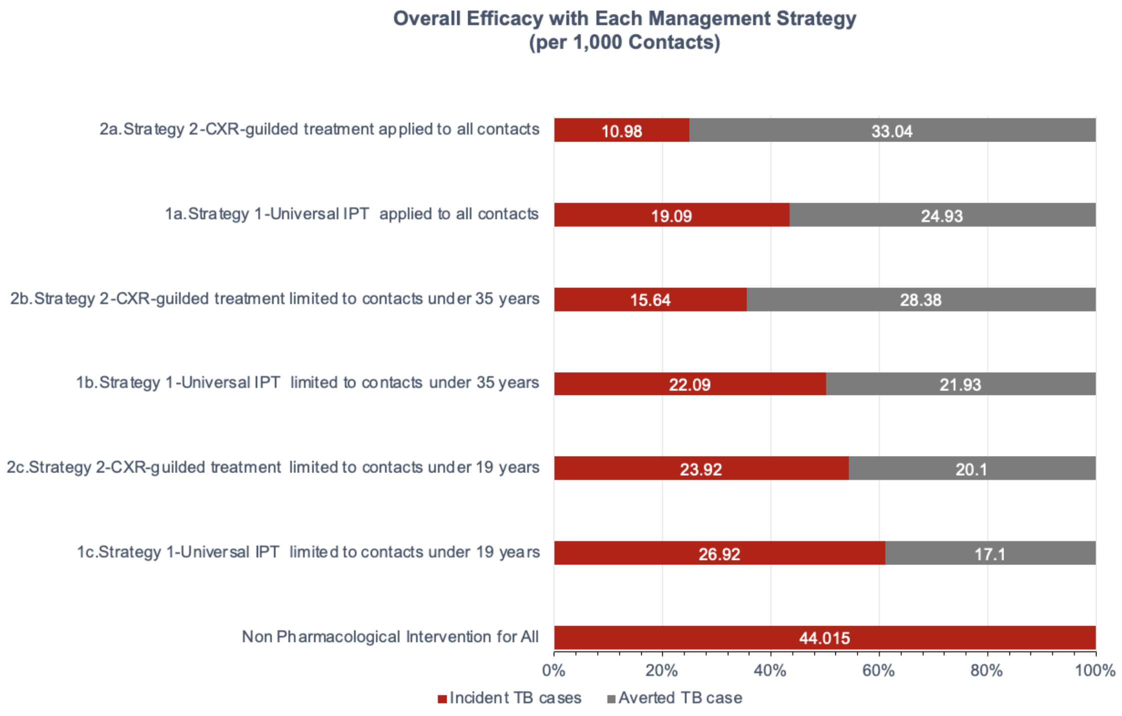

Parameter in CXR abnormality prevalence in each age group[4,5]
Parameter in age structure[4,5]
IPT= isoniazid preventive therapy.
CXR= chest-X ray
HHCs= household contacts

© 2025 Tan Q et al. *JAMA Network Open*.

eFigure 5. Comparing Outcomes of Scaling Up 2 Intervention Strategies to All Contacts When Applying 6H, 4R, and 3HP as TPT Regimen

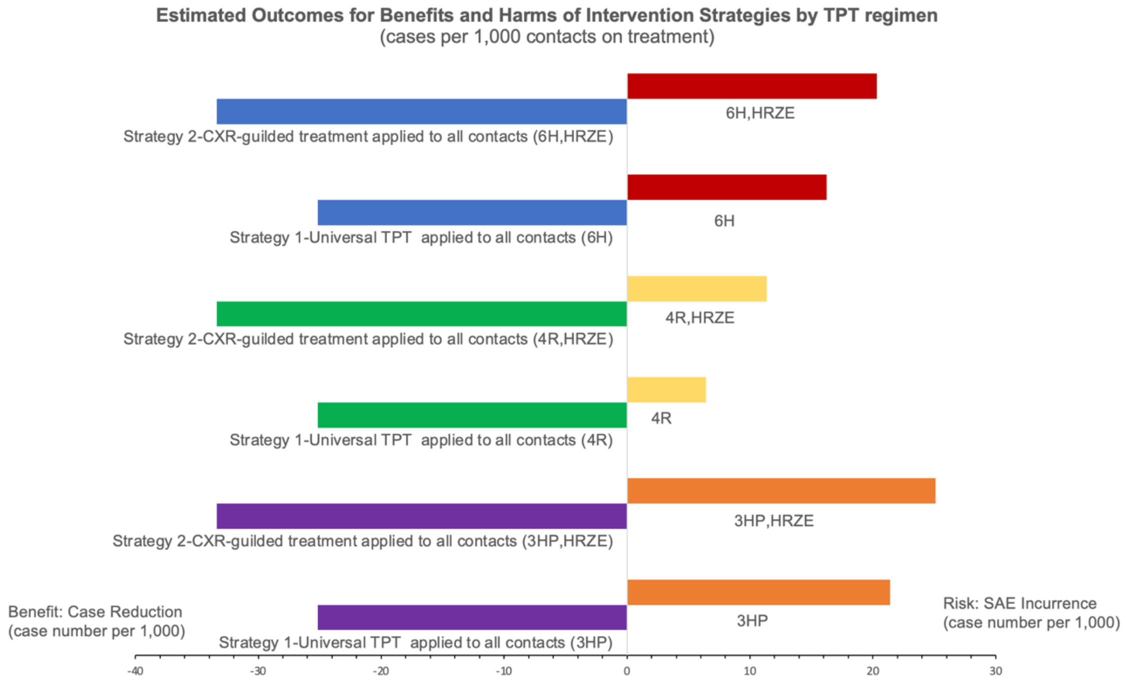

6H: 6 months of daily isoniazid
4R: 4 months of daily rifampicin
3HP: 3 months of weekly isoniazid and rifapentine
H= Isoniazid
R= Rifampin
Z=Pyrazinamide
E=Ethambutol

eFigure 6. Differences in Outcomes Between 2 Pharmacological Strategies Across Age Groups Under Varying Uptake Rate

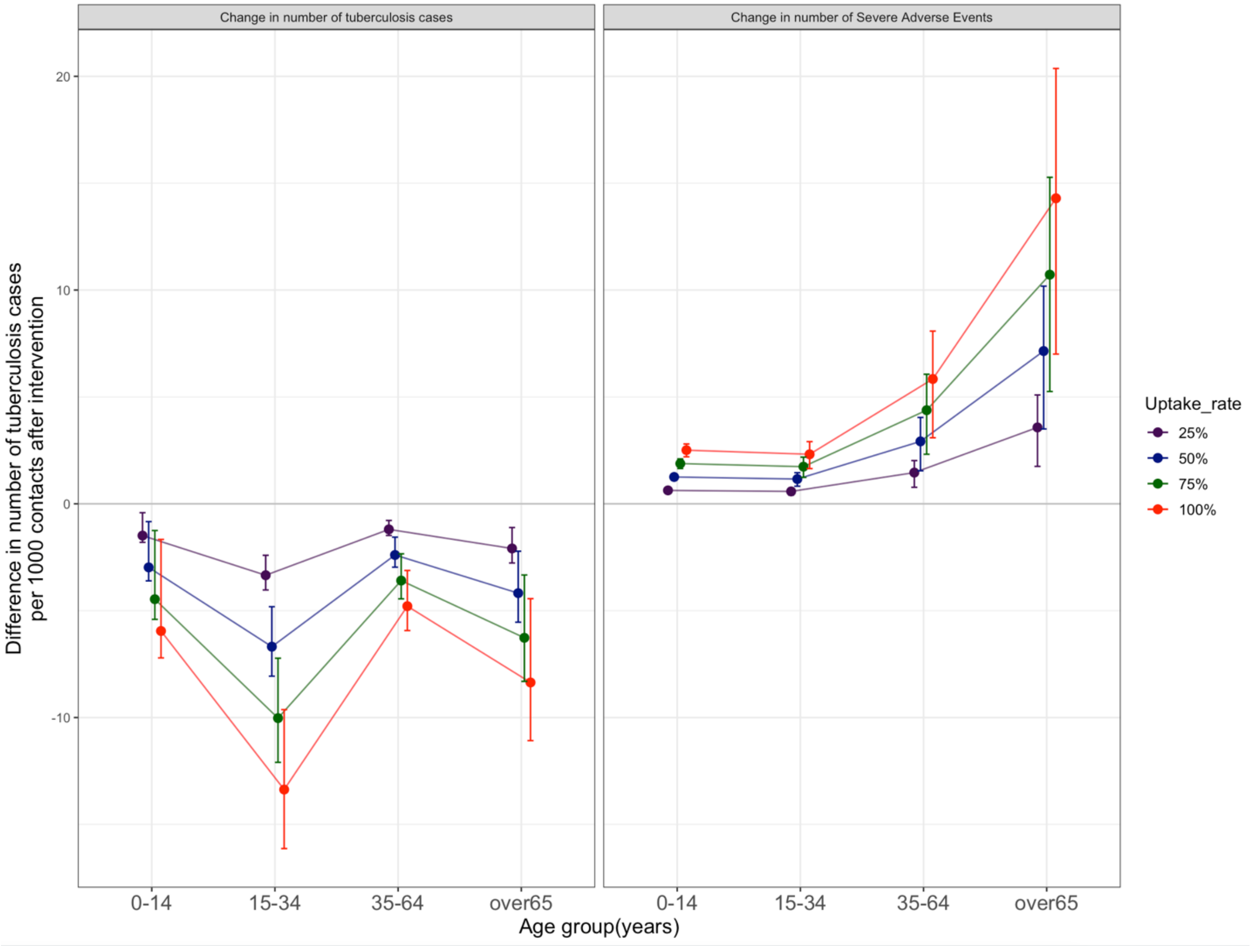

© 2025 Tan Q et al. *JAMA Network Open*.

eReferences.

1 Robert, Christian. "From Prior Information to Prior Distributions". The Bayesian Choice. New York: Springer.1994; pp. 89–136. ISBN 0-387-94296-3.

2 Orloff et al., "Conjugate Priors: Beta and Normal," math.mit.edu, retrieved October 20, 2023

3 Stevens JW. Uncertainty analysis is inherently Bayesian. Value Health. 2011;14(1):202-3

4. Huang CC, Tan Q, Becerra MC, et al. The Contribution of Chest Radiography to the Clinical Management of Children Exposed to Tuberculosis. Am J Respir Crit Care Med. Oct 01 2022;206(7):892-900. doi:10.1164/rccm.202202-0259OC

5. Tan Q, Huang CC, Becerra MC, et al. Chest Radiograph Screening for Detecting Subclinical Tuberculosis in Asymptomatic Household Contacts, Peru. Emerg Infect Dis. Jun 2024;30(6):1115-1124. doi:10.3201/eid3006.231699

6. Zenner D, Beer N, Harris RJ, Lipman MC, Stagg HR, van der Werf MJ. Treatment of Latent Tuberculosis Infection: An Updated Network Meta-analysis. Ann Intern Med. Aug 15 2017;167(4):248-255. doi:10.7326/M17-0609

7. Gray AT, Macpherson L, Carlin F, et al. Treatment for radiographically active, sputum culture-negative pulmonary tuberculosis: A systematic review and meta-analysis. PLoS One. 2023;18(11):e0293535. doi:10.1371/journal.pone.0293535

8. Turkova A, Wills GH, Wobudeya E, et al. Shorter Treatment for Nonsevere Tuberculosis in African and Indian Children. N Engl J Med. Mar 10 2022;386(10):911-922. doi:10.1056/NEJMoa2104535

9. Dorman SE, Nahid P, Kurbatova EV, et al. Four-Month Rifapentine Regimens with or without Moxifloxacin for Tuberculosis. N Engl J Med. May 06 2021;384(18):1705-1718. doi:10.1056/NEJMoa2033400

10. Prajapati AC, Shah T, Panchal S, et al. Treatment outcomes and associated factors among patients with drug-sensitive tuberculosis on daily fixed-dose combination drugs: A cohort study from Ahmedabad, India. J Family Med Prim Care. Mar 2023;12(3):452-459. doi:10.4103/jfmpc.jfmpc_1331_22

11. Menzies D, Adjobimey M, Ruslami R, et al. Four Months of Rifampin or Nine Months of Isoniazid for Latent Tuberculosis in Adults. N Engl J Med. Aug 02 2018;379(5):440-453. doi:10.1056/NEJMoa1714283

12. Campbell JR, Trajman A, Cook VJ, et al. Adverse events in adults with latent tuberculosis infection receiving daily rifampicin or isoniazid: post-hoc safety analysis of two randomised controlled trials. Lancet Infect Dis. Mar 2020;20(3):318-329. doi:10.1016/S1473-3099(19)30575-4

13. Yee D, Valiquette C, Pelletier M, Parisien I, Rocher I, Menzies D. Incidence of serious side effects from first-line antituberculosis drugs among patients treated for active tuberculosis. Am J Respir Crit Care Med. Jun 01 2003;167(11):1472-7. doi:10.1164/rccm.200206-626OC

14. Kendall EA, Shrestha S, Dowdy DW. The Epidemiological Importance of Subclinical Tuberculosis. A Critical Reappraisal. Am J Respir Crit Care Med. Jan 15 2021;203(2):168-174. doi:10.1164/rccm.202006-2394PP

15. Balcells ME, Thomas SL, Godfrey-Faussett P, Grant AD. Isoniazid preventive therapy and risk for resistant tuberculosis. Emerg Infect Dis. May 2006;12(5):744-51. doi:10.3201/eid1205.050681

16. Dean AS, Zignol M, Cabibbe AM, et al. Prevalence and genetic profiles of isoniazid resistance in tuberculosis patients: A multicountry analysis of cross-sectional data. PLoS Med. Jan 2020;17(1):e1003008. doi:10.1371/journal.pmed.1003008

17. Asencios L, Quispe N, Mendoza A, Leo E, Vasquez L, Jave O, et al. Vigilancia nacional de la resistencia a medicamentos antituberculosos, Perú 2005–2006. Rev Peru Med Exp Salud Publica. 2009;26(3):278–87 .Available from: http://www.scielo.org.pe/scielo.php?script=sci_arttext&pid=S1726-46342009000300003

© 2025 Tan Q et al. *JAMA Network Open*.
