# Supplementary material for: Integrating Early Tuberculosis States Into Contact Management in Peru
Source: JAMA Netw Open. 2025 Aug 6;8(8):e2525207. doi: 10.1001/jamanetworkopen.2025.25207 (PMC12329612; doi:10.1001/jamanetworkopen.2025.25207)
Supplement: Supplement 2. — Data Sharing Statement [file jamanetwopen-e2525207-s002.pdf]

Data Sharing Statement

Tan. Integrating Early Tuberculosis States Into Contact Management in Peru. *JAMA Netw Open*. Published August 06, 2025. doi:10.1001/jamanetworkopen.2025.25207

Data
Data available: No

Additional Information
Explanation for why data not available: Apart from the EPI cohort data, the data from the Peruvian household contact cohort study, led by Harvard Medical School and Socios en Salud in Peru, all data used in this analysis consist of publicly available data, including published clinical trials, observational studies, and meta-analyses. Dr. Megan Murray had full access to all the data in the study and takes responsibility for the integrity of the data and the accuracy of the data analysis.
